# Maternal urinary metabolic signatures of fetal growth and associated clinical and environmental factors in the INMA study

**DOI:** 10.1186/s12916-016-0706-3

**Published:** 2016-11-04

**Authors:** Léa Maitre, Cristina M. Villanueva, Matthew R. Lewis, Jesús Ibarluzea, Loreto Santa-Marina, Martine Vrijheid, Jordi Sunyer, Muireann Coen, Mireille B. Toledano

**Affiliations:** 1 Department of Epidemiology and Biostatistics, Medical Research Council–Public Health England (MRC-PHE) Centre for Environment and Health, School of Public Health, Imperial College London, W2 1PG London, UK; 2 Division of Computational and Systems Medicine, Department of Surgery and Cancer, Imperial College London, SW7 2AZ London, UK; 3 ISGlobal, Centre for Research in Environmental Epidemiology (CREAL), 08003 Barcelona, Spain; 4 Universitat Pompeu Fabra UPF, 08002 Barcelona, Spain; 5 CIBER Epidemiología y Salud Pública CIBERESP, 28029 Madrid, Spain; 6 Municipal Institute of Medical Research IMIM-Hospital del Mar, 08003 Barcelona, Spain; 7 Public Health Division of Gipuzkoa, Basque Government, 20013 San Sebastián, Spain; 8 Health Research Institute, Biodonostia, 20013 San Sebastián, Spain; 9 MRC-NIHR National Phenome Centre, Department of Surgery and Cancer, Imperial College London, IRDB Building, Du Cane Road, W12 0NN London, UK

**Keywords:** Fetal growth, Birth weight, NMR, Metabonomics, Metabolomics, In utero environment, Exposome, Pregnancy

## Abstract

**Background:**

Maternal metabolism during pregnancy is a major determinant of the intra-uterine environment and fetal outcomes. Herein, we characterize the maternal urinary metabolome throughout pregnancy to identify maternal metabolic signatures of fetal growth in two subcohorts and explain potential sources of variation in metabolic profiles based on lifestyle and clinical data.

**Methods:**

We used ^1^H nuclear magnetic resonance (NMR) spectroscopy to characterize maternal urine samples collected in the INMA birth cohort at the first (*n* = 412 and *n* = 394, respectively, in Gipuzkoa and Sabadell cohorts) and third trimesters of gestation (*n* = 417 and 469). Metabolic phenotypes that reflected longitudinal intra- and inter-individual variation were used to predict measures of fetal growth and birth weight.

**Results:**

A metabolic shift between the first and third trimesters of gestation was characterized by ^1^H NMR signals arising predominantly from steroid by-products. We identified 10 significant and reproducible metabolic associations in the third trimester with estimated fetal, birth, and placental weight in two independent subcohorts. These included branched-chain amino acids; isoleucine, valine, leucine, alanine and 3 hydroxyisobutyrate (metabolite of valine), which were associated with a significant fetal weight increase at week 34 of up to 2.4 % in Gipuzkoa (*P* < 0.005) and 1 % in Sabadell (*P* < 0.05). Other metabolites included pregnancy-related hormone by-products of estrogens and progesterone, and the methyl donor choline. We could explain a total of 48–53 % of the total variance in birth weight of which urine metabolites had an independent predictive power of 12 % adjusting for all other lifestyle/clinical factors. First trimester metabolic phenotypes could not predict reproducibly weight at later stages of development. Physical activity, as well as other modifiable lifestyle/clinical factors, such as coffee consumption, vitamin D intake, and smoking, were identified as potential sources of metabolic variation during pregnancy.

**Conclusions:**

Significant reproducible maternal urinary metabolic signatures of fetal growth and birth weight are identified for the first time and linked to modifiable lifestyle factors. This novel approach to prenatal screening, combining multiple risk factors, present a great opportunity to personalize pregnancy management and reduce newborn disease risk in later life.

**Electronic supplementary material:**

The online version of this article (doi:10.1186/s12916-016-0706-3) contains supplementary material, which is available to authorized users.

## Background

Fetal growth restriction (FGR) or excessive growth (macrosomia) affect 15 % and 10 % of all pregnancies, respectively [1, 2]. Beyond consequences at birth, abnormal fetal growth and birth weight are associated with adverse health risks in later life, for example, the development of obesity and type 2 diabetes [3, 4]. Identifying women at risk early in pregnancy has been the focus of recent prenatal care initiatives, based on maternal lifestyle factors (i.e., smoking, BMI, and diet), medical history, and a panel of serum biomarkers [5]. However, none of these methods provide high enough accuracy to detect fetal growth aberrations [6].

Exploratory metabolic profiling offers a powerful means of capturing systems-level information that reflects both maternal genetic and environmental influences, hence helping to elucidate metabolic disturbances and pathways associated with fetal outcomes [7–9]. Our recent work demonstrated the value of nuclear magnetic resonance (NMR) spectroscopic-based metabolic profiling in detecting early urinary markers (at approximately 11 weeks of gestation) of preterm birth and FGR in a nested case–control study [10]. However, longitudinal, larger-scale studies with detailed data on maternal environment, lifestyle, and medical history to characterize differential metabolic status during pregnancy are needed to identify translatable biomarkers of FGR. A recent study of healthy pregnancy with urine and blood samples at multiple time points of gestation has provided great insights into the changing pregnancy metabolome through the use of untargeted NMR [11].

To our knowledge, the present study represents the largest human investigation (with a total of 1695 metabolic phenotypes generated) in which metabolic profiling of maternal samples has been used to understand the progression of normal fetal growth. This study aimed to (1) characterize the maternal urinary metabolome throughout pregnancy, (2) identify maternal metabolic signatures of fetal growth in two subcohorts, (3) explain potential sources of variation in metabolic profiles based on lifestyle and clinical data, and finally (4) to determine the individual importance of metabolic signatures versus other maternal factors on birth weight.

## Methods

### Study population

**Fig. 1:**
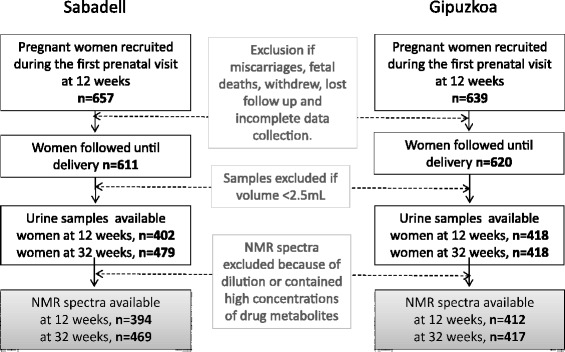
Flow chart of inclusion/exclusion of subjects and urine samples from the Gipuzkoa (GM) and Sabadell (SB) cohorts

INMA (INfancia y Medio Ambiente) is a birth cohort study in seven regions of Spain that aims to examine the role of environmental pollutants in relation to child growth and development. All participants were singleton live-born infants from two INMA subcohorts located in Gipuzkoa (Basque Country) and Sabadell (Catalonia) [12]. The women were interviewed twice during pregnancy (in the first and third trimesters of gestation) to obtain information about their sociodemographic characteristics and lifestyle variables. The urine samples were collected in the same interview in the morning (spot samples). Urine was collected in 100 mL polyethylene containers and stored at −20 °C. One aliquot of the sample from each of the participants was sent to the laboratories of the Department of Surgery and Cancer, Imperial College London, UK, to be analyzed. NMR spectra of urine were generated from 412 and 417 subjects for the first and third trimesters, respectively (12.4 ± 1.2 and 33.9 ± 1.3 weeks, respectively) in Gipuzkoa and from 394 and 469 subjects for Sabadell (Fig. 1). The INMA project was approved by the Ethical Committees of the participating centres, and all subjects gave written consent at enrolment and delivery.

### Definition of the fetal and birth measurements

Fetal growth scores or standard deviation scores (z-scores) were obtained using longitudinal growth curves calculated for each individual adjusting for constitutional factors known to affect fetal growth (i.e., maternal age, height, parity, pre-pregnancy weight, country of origin, father’s height, and fetal sex). Unconditional z-scores at a certain time point describe the size of a fetus at this time and conditional z-scores describe the growth of a fetus during the respective time interval, i.e., at 12–20, 20–34, and 12–34 weeks of gestation [13]. Anthropometric measures at birth included body weight and placental weight and were scaled to z-scores by subtracting the mean and dividing by the standard deviation. For further details on phenotype measurements and covariate definitions, such as gestational age, see Additional file 1: Supplementary Methods and a previous article on the INMA cohort [13].

### Metabolic profiling by NMR

Urinary metabolic profiles were generated using ^1^H NMR spectroscopy (Additional file 1: Supplementary Methods) [14]. Fully resolved spectral data corrected for different dilution in spot urine samples (probabilistic quotient normalization) were used to model metabolic variation across the two time points of pregnancy whereas the prediction of fetal growth measures was based on 64 semi-quantified metabolites after manual spectral binning. These metabolites, represented by spectral integrals of single representative resonances, were selected on the basis of being present in a high proportion of the spectra, having a high signal-to-noise ratio, and exhibiting limited overlap with other resonances. A list of the 64 metabolites (of which 47 were fully identified), their integration window, their metabolic clusters, and details on assignments are presented in Additional file 2: Table S1. Metabolite correlation clusters were created using the complete linkage method for hierarchical clustering in order to present metabolite-outcome associations according to urinary metabolite clusters (heatmaps shown in Additional file 3: Figure S1, Additional file 4: Figure S2, Additional file 5: Figure S3, and Additional file 6: Figure S4). Metabolite inter-correlation heatmaps were also used to ensure that selected resonances from unassigned metabolites arise from unique metabolites (i.e., no consistent patterns across unassigned metabolites across the two cohorts and time points).

### Statistical analysis

#### Step 1. Identify main sources of metabolic variation between first and third trimesters of gestation

Metabolic profiles, as digitized spectra, were subjected to exploratory analysis where multivariate projection methods such as principal component analysis (PCA) provided an overview of the data and helped identify the main sources of biological and technical variation and potential outliers. Differences between first and third trimester samples were further explored through the application of orthogonal-partial least squares discriminatory analysis (O-PLS-DA). In order to avoid over-fitting the data, a 7-fold cross-validation was used and statistical parameters (R^2^Y and Q^2^Y representing the goodness of fit and predictive ability) were calculated. All multivariate statistical analyses were performed using MATLAB.

#### Step 2. Identify metabolites associated with fetal growth

For each metabolite in each subcohort, multivariate linear regression analysis was performed to estimate metabolite-fetal growth associations whilst controlling for the time of sampling. Each metabolite integral was log-transformed (log base 10) and modelled separately. Coefficients from the regression models were multiplied by the logarithm of 1.5 to derive an effect estimate for a 50 % increase in metabolite levels [15]. Regression coefficients are presented as a percent of change in the z-score of each fetal growth measure.

#### Step 3. Assess the extent to which the metabolite panel associated with fetal growth can be explained by known growth-related factors from epidemiological and clinical data

We considered the following growth-related parameters: maternal clinical parameters, maternal lifestyle in third trimester, dietary intake in third trimester and sociodemographic characteristics of both parents, all of which have been previously reported to be strongly associated with birth weight (38 parameters selected, full details in Additional file 7: Table S2). Pairwise spearman correlation among metabolites and maternal parameters are represented as circos plots, where each line/link is represented only for correlations with an adjusted *P* value under 0.05 (*P* adjusted for by FDR using the function “*P*-adjust” in R). In addition, correlation adjusted scores (CAR scores) were used to measure the correlation between metabolite levels and all the Mahalanobis-decorrelated predictors in one model (growth-related factors selected from epidemiological and clinical data) as implemented in the R-package ‘care’ [16]. The correlation shrinkage intensity lambda was 0, i.e., we basically used the empirical correlation structure to estimate the variable covariance. To quantify the explained variance assignable to the different explanatory variables separately, we summarized squared CAR scores separately and multiplied them by the sign of the correlation coefficient to interpret the direction of association. Continuous predictors were transformed in the case of non-normal distribution. A summary of the maternal predictors analyzed can be found in Additional file 7: Table S2.

#### Step 4. Determinants of birth weight: a variance decomposition analysis

In order to estimate the importance of different pregnancy determinants of birth weight, including the third trimester urinary metabolite panel selected at step 2, we again used CAR scores. *P* values for empirical CAR scores were computed. We summarized separately squared CAR scores and their sum into groups of variables (variation attributed to metabolites, clinical biochemistry, diet, lifestyle, and sociodemographic parameters).

## Results

A selection of baseline characteristics of the study participants are presented in Table 1 and comprehensive lifestyle, clinical, and dietary variables in Additional file 7: Table S2. Mothers were 28–33 years old (interquartile range) and predominantly Spanish (85–90 %). Women in Gipuzkoa were more educated, from a higher social class, and generally healthier than those among the Sabadell subcohort (based on lower body mass index, more physical activity, and better general health, for details see Additional file 7: Table S2).

**Table 1:** Baseline characteristics of the Gipuzkoa and Sabadell subcohorts. Count and mean values are presented (percentage and standard deviation in parenthesis)

	Gipuzkoa (*n* = 419)^a^	Sabadell first trimester (*n* = 394)	Sabadell third trimester (*n* = 469)	*P* ^b^
Maternal education: Primary or without education	56 (13.4 %)	108 (27.5 %)	108 (27.5 %)	
Secondary	147 (35.1 %)	160 (40.6 %)	160 (40.6 %)	***
University	216 (51.6 %)	123 (31.2 %)	123 (31.2 %)	***
Small for gestational age	34 (8.1 %)	45 (11.4 %)	45 (11.4 %)	***
Preterm birth (<37 weeks)	8 (1.9 %)	11 (2.8 %)	11 (2.8 %)	
Parity:nulliparous	231 (55.1)	219 (55.5)	261 (55.6)	
Child sex: Males	218 (52.0 %)	197 (50.0 %)	241 (51.4 %)	*
Body mass index before pregnancy: < 18.5 (Underweight)	12 (2.9 %)	24 (6.1 %)	27 (5.8 %)	**
18.5–25 (Healthy)	320 (76.4 %)	268 (68 %)	310 (66.1 %)	
25.1–30 (Overweight)	57 (13.6 %)	72 (18.3 %)	93 (19.8 %)	
>30 (Obese)	30 (7.2 %)	30 (7.6 %)	39 (8.3 %)	
Birth weight (g)	3310 (440)	3240 (410)	3260 (420)	
Gestational week at birth	39.7 (1.3)	39.7 (1.4)	39.7 (1.4)	

^a^Since the population in Gipuzkoa subcohorts at weeks 12 and 34 were almost identical (only 5 women are different in the third trimester), characteristics are presented for the combined subcohorts at weeks 12 and 34 for a total of 419 women

^b^ *P* values were calculated using the χ^2^ test (categorical variables) or Mann–Whitney test (continuous) between Sabadell women (week 34 subcohort) and Gipuzkoa women. Numbers do not add because of missing values in variables (presented in Additional file 3)

Legend: * *p*-value<0.05; ** *p*-value<0.01; ***p-value<0.001

### Characterization of maternal metabolic shift during pregnancy

**Fig. 2:**
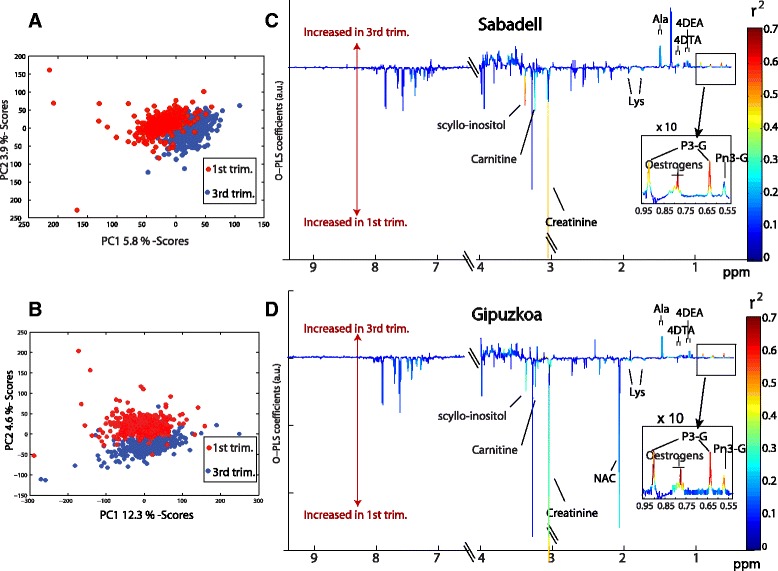
Metabolic differences in first and third trimesters of pregnancy in urinary NMR spectral profiles. Principal component analysis (PCA) and orthogonal-projection to least squares-discriminant analysis (O-PLS-DA) models discriminating PCA score plots for the Sabadell (**a**) and Gipuzkoa subcohorts (**b**) at first (blue) and third trimesters (red). **c** O-PLS-DA loadings coefficient plot in the Sabadell subcohort. The height of spectral peaks represents the covariance, and the color code corresponds to the coefficient of determination, r^2^. Model statistics: R^2^Y = 0.88, R^2^X = 0.22, Q^2^Y = 0.84. **d** Same as (**c**) for Gipuzkoa subcohort, R^2^Y = 0.91, R^2^X = 0.13, Q^2^Y = 0.86. *4-DEA* 4-deoxyerythronic acid, *4-DTA* 4-deoxythreonic acid, *Ala* alanine, *Lys* lysine, *NAC* N-acetylneuraminic acid **d**, *P3G*, 5β-pregnane-3α,20α-diol-3α-glucuronide,* Pn3-G*, a progesterone metabolite (likely allopregnanolone and isomers)

An overview of the NMR spectral data in the Sabadell and Gipuzkoa subcohorts, using dimension reduction regression methods (PCA and O-PLS-DA models) after normalization for sample dilution, provided a clear separation of the two trimesters sampled during pregnancy (Fig. 2a, b). This separation occurred in the first and second principal components in the PCA model (in Sabadell, PC1: R^2^X = 13.5 %, PC2: R^2^X = 5 %, in Gipuzkoa, PC1: R^2^X = 5.8 %, PC2: R^2^X = 3.9 %). Discriminatory metabolites were identified from the O-PLS-DA coefficients (Fig. 2c, d) on the basis of the regression coefficient strength (Model statistics: R^2^Y = 0.88, R^2^X = 0.22, Q^2^Y = 0.84 for Gipuzkoa subcohort, R^2^Y = 0.91, R^2^X = 0.13, Q^2^Y = 0.86 for Sabadell subcohort). Steroid hormone by-products increased by 3-fold in the third trimester, including 5β-pregnane-3α,20α-diol-3α-glucuronide (P3G, progesterone by product), a progesterone metabolite (likely allopregnanolone and isomers), and a mixture of estrogen metabolites which had previously been uncharacterized in ^1^H NMR spectra of maternal urine. The identity of P3G was confirmed by chromatographic isolation of the target feature (Additional file 1: Supplementary Methods) and comparison of MS/MS spectra to an authentic reference compound in accordance with reported guidelines for metabolite identification [17]. In addition, creatinine, carnitine, and scyllo-inositol were significantly decreased in third trimester samples, whereas alanine and 4-deoxyerythronic acid were increased.

### Maternal urinary metabolic phenotype associated with greater fetal growth

**Fig. 3:**
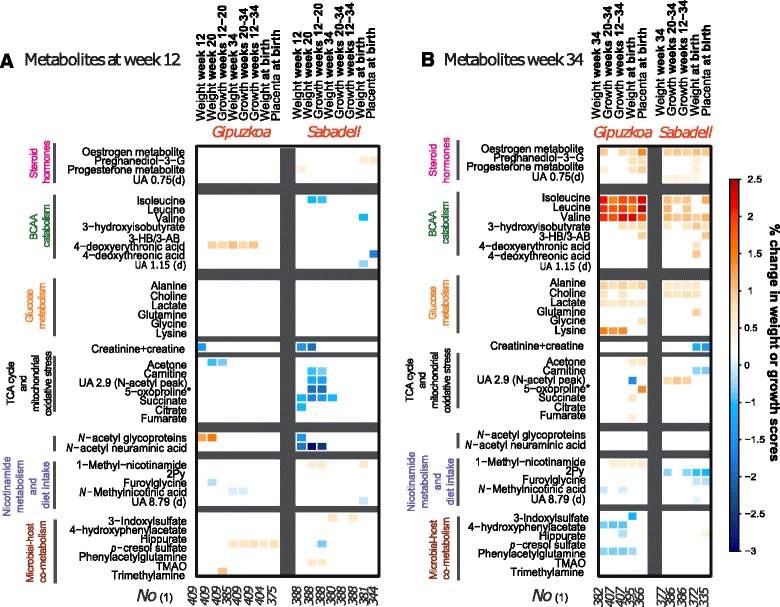
Associations between first (**a**) and third (**b**) trimester maternal urinary metabolites and fetal growth scores in the Gipuzkoa and Sabadell subcohorts. Percent of change in the standard deviation scores of the fetal growth measure (customized growth curve for each fetus) are presented as a heat map where the colour scale corresponds to the strength of the change for an increase of 50 % in the metabolite spectral integral. Only metabolites which were significantly associated with fetal growth in both subcohorts and with a *P* value < 5 % are presented. All the models were adjusted for the time of sampling during gestation and models for birth weight and placental weight were further adjusted for maternal weight and height, father’s height, parity, child’s sex, and gestational age at sampling and at birth. (1) Number of individuals included in the adjusted models, that is, number without missing values for any variable included in each model. *Tentative assignment. *2Py* *N-methyl-* 2-pyridone-5-carboxamide, *3-HBA/3-ABA* *3-hydroxybutyrate/3-aminoisobutyrate*, UA unassigned, *d* doublet, *Pregnanediol-3-G* 5β-pregnane-3α20α-diol-3α-glucuronide, *TMAO* Trimethylamine oxide. Weeks 12, 20, and 34 stand for the gestational week at which the fetal growth outcome was calculated or the conditional growth between these weeks

Based on longitudinal fetal ultrasound measurements and birth weight, fetal growth was assessed (estimated fetal weight based on measures of abdominal circumference, biparietal diameter and femur length). Of 64 urinary metabolites present during the third trimester, 10 were associated in both subcohorts with greater growth (with *P* < 0.05) at 12–34, 20–34, and 34 weeks of gestation and birth weight (Fig. 3b). Levels of branched-chain amino acids (BCAAs) isoleucine, valine, leucine, and 3 hydroxyisobutyrate (3-HIB, a metabolite of valine) were associated with a significant weight increase at week 34 of up to 2.4 % in Gipuzkoa (*P* < 0.005) and 1 % in Sabadell (*P* < 0.05). Similarly, estrogens were consistently associated with greater fetal growth between 12–34 and 20–34 weeks and fetal weight at week 34 and at birth (*P* = 0.007 in Gipuzkoa and 3.10^-5^ in Sabadell). Progesterone by-products, were solely associated with anthropometric measures at birth in contrast to estrogens, with a 0.3–0.6 % associated increase in birth weight (*P* = 0.001–0.02). BCAAs, estrogens and, to a lesser extent, progesterone by-products were found to be consistently correlated to each other suggesting a common source of biological variation. Other metabolites involved in glucose metabolism, including alanine and choline, presented similar trends with fetal weight outcomes. 3-hydroxybutyrate/3-aminoisobutyrate were significantly associated in both subcohorts with increased placental weight (+0.9 %).

Using first trimester metabolic profiles, only creatinine/creatine were associated in both subcohorts with fetal growth with a 1.2 % change in weight at 12 weeks (*P* = 0.027 and 0.011 in Gipuzkoa and Sabadell, respectively; Fig. 3a). In the Sabadell women, a panel of maternal urinary metabolites at 12 weeks, including succinate, citrate, carnitine and 5-oxoproline, were consistently negatively associated with early fetal growth, at weeks 12–20, leading to decreased body weight at week 20. There were no consistent associations between first trimester urinary metabolic phenotypes and fetal weight in the third trimester or at birth. In an additional analysis, we also found that fetal weight estimates at 12 and 20 weeks of gestation were poor predictors of birth weight (spearman *r* < 0.1).

### Environmental, lifestyle, and clinical markers associated with maternal metabolic signatures of fetal growth

**Fig. 4:**
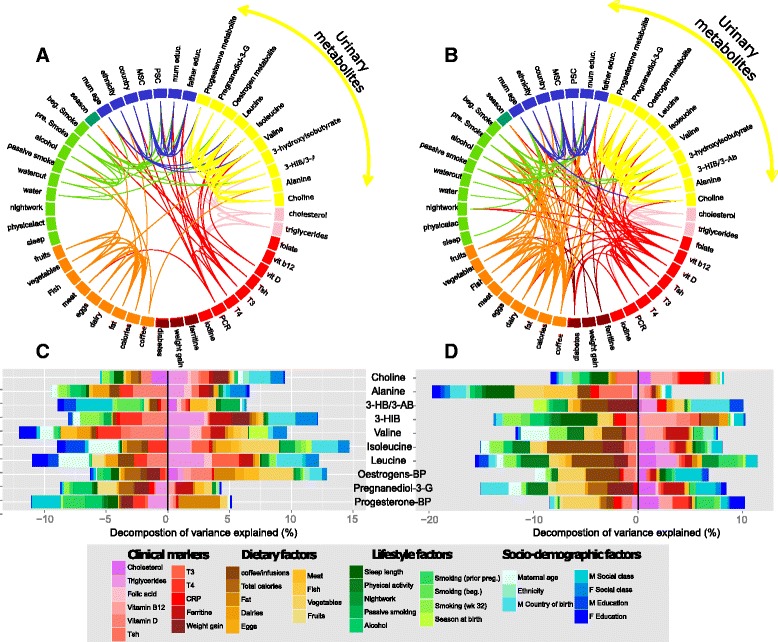
Clinical and lifestyle influences on metabolite signatures of fetal growth measured in third trimester urine samples. **a** and **b** Correlation networks in Gipuzkoa (left) and Sabadell (right) between maternal and paternal determinants of fetal growth, including selected urinary metabolites. **c** and **d** Decomposition of variance analysis of metabolite signatures of fetal growth based on cohort data available in most participants. *3-HBA/3-ABA* 3-hydroxybutyrate/3-aminoisobutyrate, *3-HIB* 3-hydroxyisobutyrate, *BP* by-product, *CRP* C-reactive protein, *M* maternal, *P* Paternal, *Pregnanediol-3-G* 5β-Pregnane-3α,20α-diol-3α-glucuronide, *T3* triiodothyronine, *T4* thyroxine, *TSH* thyroid stimulating hormone

Pairwise correlation across determinants of fetal growth and our panel of maternal urinary metabolites associated with fetal growth in the third trimester, revealed significant associations. Determinants of fetal growth included constitutional factors (parental anthropometry, parity, and sex), lifestyle (sleep, physical activity, night work, daily intake of alcohol (g), active and passive smoking, dietary intake), sociodemographic and clinical markers (lipids, vitamins, thyroid hormones, ferritin, C reactive protein, rate of weight gain during pregnancy). Represented as yellow branches on the circos plot, metabolite levels were inter-related with clinical markers (thyroid T3 hormone, vitamin D, and triglycerides) and lifestyle factors, in particular smoking (Fig. 3a, b for Gipuzkoa and Sabadell, respectively). The strong associations between parental socioeconomic status and adverse lifestyle exposures (in green) are also evident from the plot. Analysis of these metabolic signatures showed that up to 16–28 % of their observed variation can be explained by a combination of maternal environmental and clinical factors (adjusted R^2^ score using shrinkage methods, Fig. 4c, d and full Table in Additional file 8: Table S3). Triglycerides and cholesterol explained up to 5 % of the variation observed in urinary BCAAs and progesterone by-products in both subcohorts (purple key in Fig. 4c, d). Intake of coffee/tea (brown key in Fig. 4) in the third trimester was the most important dietary factor in Sabadell associated with reduced levels of estrogens, leucine, isoleucine and, to a lesser extent, valine. Physical activity was also associated with reduced levels of P3G and BCAAs (dark green key in Fig. 4). This result appeared only in Sabadell, where women reduced their activity dramatically in the third trimester with the majority partaking in little physical activity (45 %) compared to Gipuzkoa (30 %) (*P* < 0.001). Variation in vitamin D (serum 25-hydroxyvitamin) and thyroid hormones (T3) were also related to levels of BCAAs, 3-HIB, alanine, and choline (only in Gipuzkoa). Sabadell first trimester metabolite levels associated with reduced fetal weight at weeks 12 and 20 (including isoleucine, leucine, creatinine/creatine, carnitine) were specifically explained by sociodemographic factors (up to 8 % of variance explained) and smoking exposures (Additional file 9: Figure S5).

### Predictive power of third trimester metabolic signatures and other maternal factors on birth weight

**Fig. 5:**
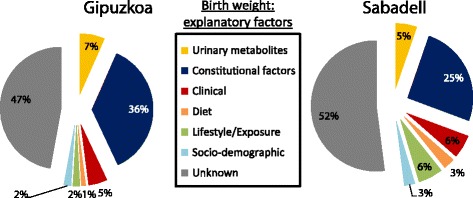
Determinants of birth weight. Variance decomposition analysis of factors affecting birth weight in two subcohorts, in Gipuzkoa and Sabadell. Constitutional factors include by mother’s weight, pre-pregnancy, mother’s height, father’s height, parity, gestational age at birth, newborn sex

Taken together, the combined explanatory power of birth weight from all data available in this study (including maternal urinary metabolites, lifestyle factors measured in the third trimester, clinical markers measured in the first trimester, and constitutional factors) was 48 % and 53% in Sabadell and Gipuzkoa, respectively (adjusting for inter-correlated predictors; Fig. 5, for details see Additional file 8: Table S3). The panel of 10 urinary metabolites in the third trimester, which were selected based on their significant reproducible associations with fetal growth, explained 12 % of this total known variance in birth weight in Sabadell and Gipuzkoa, respectively, adjusting for all other factors measured in this study, including important constitutional factors. This is of comparable or larger magnitude than the variance explained by each of clinical, dietary, and lifestyle factors, all traditionally considered important in the etiology of fetal growth (Fig. 5).

## Discussion

Through the use of two independent birth subcohorts we have identified, for the first time, reproducible maternal urinary metabolic signatures of fetal growth and birth weight on the largest scale to date. We have demonstrated novel and significant relationships between steroid hormones, BCAAs, alanine, and choline with fetal weight at 34 weeks of gestation and birth weight which combined accounted for 12 % of the variation in birth weight that could be explained after adjustment for all other lifestyle, clinical, and constitutional factors. Moreover, we have quantified these novel relationships, showing that a 50 % increase in isoleucine, leucine, and valine is each associated with a 1–2.4 % (equivalent to 5–11 g deviation from the expected mean weight, based on the whole population standard deviation) increase in birth weight. Through analysis of rich data on lifestyle and clinical markers, we have demonstrated further understanding of the inter-relationships between fetal/birth weight and maternal urinary metabolites.

A large proportion of the metabolites measured were affected by the time of gestation but steroid by-products of progesterone and estrogens were particularly increased between week 12 and 34 (ca. 3-fold). Surprisingly, our study was the first to confirm the identity of these metabolites via ^1^H NMR despite previously being identified as important urinary markers during pregnancy [18–20]. According to previous publications, 22 % of progesterone metabolites are excreted in the urine as 5β-pregnane-3α,20α-diol-3α-glucuronide [21, 22], which corroborate our LC-MS/MS and NMR results. Circulating amino acids are known to decrease during pregnancy, partially due to hormonal changes,  a high demand in essential amino acids by the fetus, and renal changes [23]. This phenomenon was observed in our data, including for alanine, glycine, and BCAAs such as isoleucine and leucine, which increased by 10–20 % in the third trimester urine compared to first trimester. This can also be explained by a decreased maternal BCAA oxidation during pregnancy in order to improve BCAA availability for the fetus [24]. In addition, carnitine levels were 2-fold lower later in gestation, which is consistent with characteristic enhanced lipid oxidation later in pregnancy, in agreement with previous reports [18, 25].

BCAAs are essential nutrients that cannot be synthesized* de novo* in humans. Their homeostasis is therefore maintained by degradation and dietary intake only. BCAAs are potentially particularly important during gestation as an energy source, or utilized for biosynthetic purposes. They can be oxidized to keto-acids, which are in turn decarboxylated to form acetyl-CoA and succinyl-CoA, which eventually enter the Krebs cycle (TCA cycle) and produce ATP. BCAA oxidation is particularly important in cases of fasting or even in patients with type 2 diabetes and obesity, where there is a switch from glucose to other substrates such as ketone bodies, glucogenic amino acids, and fatty acids for energy production [26]. Some studies using metabolomics have also demonstrated elevated levels of BCAAs in pregnant mothers with gestational diabetes compared to normal pregnancies [27, 28]. Previous metabonomic studies corroborate our results, although on a smaller sample scale, where a general decrease of amino acids in maternal urine at gestational weeks 14–26 was observed in FGR cases (*n* = 10) compared to controls (*n* = 84) [9]. Significant differences in BCAA levels, along with other circulating amino acids, were also identified in plasma and cord blood studies of IUGR babies [8, 29]. The importance of BCAAs as predictive markers of gestational diabetes but also in non-pregnant populations of adiposity and type 2 diabetes is well established [27, 28, 30]. Possible mechanisms involve 3-HIB as a cross-regulatory signal between the catabolism of BCAAs and endothelial fatty acid uptake. Indeed, excess catabolic flux of BCAAs, as observed in diabetes patients and in our study in the third trimester for those with greater fetal weight, can promote lipid accumulation and glucose intolerance [31]. We also found evidence that physical activity during the third trimester was significantly associated with lower levels of BCAAs, which corroborate results from a recent study on obesity suggesting that exercise increases the utilization of leucine in muscle and prevents hyperaminoacidemia associated with lack of exercise and insulin resistance [30]. Physical activity, as well as other modifiable lifestyle/clinical factors, such as coffee consumption, vitamin D intake, or smoking, could be the target of interventions to help women to maintain appropriate BCAA metabolism and consequently improve fetal growth outcomes. Overall, Sabadell women had less favorable environmental conditions compared to Gipuzkoa, in particular in terms of dietary supplementation, smoking exposure, surrounding greenness, chemical exposure, and general lifestyle, which were related to subsequent poorer clinical outcomes [32–34], and may be related to the unique 1st trimester metabolic phenotype associated with fetal growth.

Urinary metabolites of steroid hormones were positively associated with fetal growth in the late stages of pregnancy in our study, which corroborate previous findings [35]. Starting at the third month of pregnancy, these hormones are mainly produced by the placenta and then metabolized by fetal and maternal adrenal glands, placenta, and fetal and maternal liver [36]. Therefore, the hormone by-products measurable in the urine, usually the steroid soluble fraction, are the products of the interaction of the maternal–fetal–placental unit [37]. Previous reports, which corroborate our results, found that progesterone and estradiol were reduced in FGR cases (hence increased for newborns with greater birth weight, as observed in our study) when measured in maternal blood in the third trimester [35]. By-products of progesterone were also found decreased in metabonomics studies of poor perinatal outcomes using a LC-MS platform [38, 39]. It was also shown via NMR metabonomics on maternal urine and plasma collected at different time points of pregnancy that urine signals arising from progesterone metabolites (0.63 and 0.56 ppm) were correlated with increases in plasma high-density lipoprotein and low- and very low-density lipoprotein throughout pregnancy, confirming the role of these steroid metabolites in lipoprotein/protein metabolism during pregnancy [20].

First trimester metabolic phenotypes could mainly predict fetal weight at weeks 12 and 20, but not at third trimester and birth and with little consistency across the two populations. In Sabadell, metabolites related to (1) mitochondrial processes such as energy production through the TCA cycle or lipid metabolism, including for succinate, citrate, and carnitine, or (2) to oxidative stress were negatively associated with fetal weight and growth but also correlated to sociodemographic status and smoking. Renal changes and inflammation may be at the origin of some of the markers of early fetal growth impairment observed such as excreted creatine/creatinine (validated in both cohorts), 5-oxoproline, carnitine, and *N-*acetylneuraminic acid. Creatinine is the breakdown product of creatine and creatine phosphate, which are amino acid derivatives involved with cellular energy production, principally in muscles. Creatinine excretion during pregnancy was shown to decrease, as also observed in this study, as well as levels in serum due to increased glomerular filtration rate [23]. Interestingly, creatine supplementation during pregnancy was recently proposed to have benefits for the fetus and neonate whenever oxidative stress or feto-placental hypoxia arise [40]. Previous assessment of early fetal growth using multiple ultrasound measures found that size at birth does not correspond to fetal growth rate, as confirmed in our data [41]. Early events during fetal development may not result in a visible phenotype at birth due to catch-up growth during the third trimester, but may influence health outcomes during childhood and adult life [41, 42]. There is evidence to suggest that first trimester growth correlates with cardiovascular risk factors in school age children even after adjusting for birth weight [42]. An understanding of the impact of early fetal growth impairment or acceleration on childhood outcomes requires further investigations.

Our study benefitted from the use of two independent subcohorts for validation, which gives a realistic representation of the population at large in comparison with matched case–control studies that aim to minimize population homogeneity. The use of longitudinal ultrasound measurements and individualized growth curves for measuring fetal growth enabled several potentially critical windows throughout pregnancy to be explored. We note some study limitations. We have concentrated on the application of untargeted NMR spectroscopic-based profiling. This approach is limited with respect to its analytical sensitivity and spectral overlap of metabolite resonances, which was apparent for the steroid hormones. However, we successfully integrated the NMR spectroscopic analysis with HPLC fractionation and UPLC-MS in order to annotate a number of steroid hormones. Our work could be complemented with LC-MS-based platforms to enhance overall coverage as well as quantification of targeted classes of metabolites. This study also relied on spot measurements of urine, which display greater intra-individual variability in comparison to 24-h urine collection or first morning voids. We have identified novel inter-relationships between fetal/birth weight, metabolites, and lifestyle/clinical factors, but the cross-sectional nature of these data do not allow further causal relationships to be inferred. Rather, these findings capture the metabolic signatures of a myriad of physiological (both maternal and fetal), genetic, environmental, and other lifestyle characteristics associated with fetal growth, but not one individual lifestyle/clinical parameter could largely explain the variation of a single metabolite.

Different statistical frameworks were used to define the first and third trimester differences, assumed to have a strong systematic effect on the maternal urine metabolome, and to define metabolite to clinical growth data relationships, which were assumed to be more subtle in a free living population. More specifically, multiple linear regression was chosen for step 2 (metabolite to clinical data relationships) because of the ease of interpretation of multiple linear regression and to enable adjustment for confounding factors, including sampling time and birth-related variables. In particular, it was important to be able to quantify the impact of metabolite levels on growth z-score.

The first scan visit at 11–14 weeks is the most pertinent clinical screening for high-risk pregnancies that would benefit from personalized management. The addition of metabolic markers to clinical assessment could provide further information on the physiological status of the pregnant mothers, in particular in case of failure of placental function due to oxidative stress. Up to now, no single biomarker was found to be predictive and specific enough for abnormal fetal growth (FGR or macrosomia) for clinical use, including in this paper. Clinical assessment may benefit from a combination of markers, including Doppler ultrasonography [43] and algorithms, computed taking into account previous pregnancy history, ethnicity, and baseline lifestyle factors, and perhaps even environmental exposures. These algorithms should also be flexible to include any local specificity in cases, for instance, where there is widespread vitamin D deficiency or a lack/excess of iodine supplementation. Intervention in high risk pregnancies is an important area currently being developed, as well as personalized programs for pregnant mothers. Several metabolic pathways identified in this paper, and already tested in animal models as exerting control over fetal growth, could be tested in clinical trials to evaluate their safety and efficacy. Interestingly, melatonin, carnitine, and creatine are currently being tested for their anti-oxidant properties [40, 44, 45]. Due to the potent growth promoting effects of BCAAs in the third trimester, maternal protein supplementation in pregnancies at risk of FGR to improve fetal growth remains an attractive option. For example, dietary isoleucine was found to significantly reduce the degree of growth retardation normally observed in fetuses from pregnant rats fed a phenylketonuria-inducing diet [46]. However, adverse fetal outcomes observed in several clinical studies highlight the need to fully understand the mechanisms by which additional amino acids in the maternal diet are transferred to the fetus and how the fetus handles the protein load [47].

## Conclusion

The present study represents the largest human investigation (n > 800) in which non-targeted proton NMR spectroscopy has been used to understand the changes in urinary metabolic phenotypes over the course of pregnancy in two independent Spanish populations. We identified, for the first time, 10 maternal urinary metabolites predictive of fetal growth using longitudinal data and birth weight, including BCAAs and steroid hormone by-products. We could explain a total of 48–53 % of the total variance in birth weight, of which urine metabolites had an independent predictive power of 12 % adjusting for all other lifestyle/clinical factors. These results highlight prenatal maternal modifiable factors, i.e., metabolic phenotypes that are associated with fetal development. The current study identified metabolic markers in the third trimester, and the metabolic pathways identified, in particular BCAAs, could be the subject of future investigations in humans. This novel approach to prenatal screening, combining multiple risk factors, presents a great opportunity to personalize pregnancy management and potentially reduce disease risk in the later life of the newborn.

## Additional files

Additional file 1: Supplementary methods. (DOCX 38 kb)
Additional file 2: Table S1. List of metabolite integrals and assignments obtained from NMR urine spectra. Abbreviations: UA, unassigned. *The method of multiple spike-in of authentic standards in the original sample allowed to validate the identity of NMR signals. The identity of P3G was confirmed by chromatographic isolation of the target feature (SI Methods) and comparison of MS/MS spectra to an authentic reference compound in accordance with reported guidelines for metabolite identification (Sumner et al. 2014). **Citation for STORM: Subset Optimization by Reference Matching (STORM): An Optimized Statistical Approach for Recovery of Metabolic Biomarker Structural Information from 1H NMR Spectra of Biofluids. Joram M. Posma, Isabel Garcia-Perez, Maria De Iorio, John C. Lindon, Paul Elliott, Elaine Holmes, Timothy M. D. Ebbels, and Jeremy K. Nicholson. Analytical Chemistry 2012 84 (24), 10694-10701 DOI: 10.1021/ac302360v. (DOCX 43 kb)
Additional file 3: Figure S1. Heatmap of metabolite inter-correlation in urinary ^1^H NMR spectral profiles from Gipuzkoa at week 12 of gestation (metabolite order created using the complete linkage method for hierarchical clustering). (PDF 48 kb)
Additional file 4: Figure S2. Heatmap of metabolite inter-correlation in urinary ^1^H NMR spectral profiles from Gipuzkoa at week 34 of gestation (metabolite order created using the complete linkage method for hierarchical clustering). (PDF 47 kb)
Additional file 5: Figure S3. Heatmap of metabolite inter-correlation in urinary ^1^H NMR spectral profiles from Sabadell at week 12 of gestation (metabolite order created using the complete linkage method for hierarchical clustering). (PDF 48 kb)
Additional file 6: Figure S4. Heatmap of metabolite inter-correlation in urinary ^1^H NMR spectral profiles from Sabadell at week 34 of gestation (metabolite order created using the complete linkage method for hierarchical clustering). (PDF 48 kb)
Additional file 7: Table S2. Characteristics of women in Gipuzkoa and Sabadell during pregnancy included in theanalysis. Since the population characteristics in the Gipuzkoa cohort are almost identical between the first and third trimesters (only 5 women are different in the third trimester), the table is combined for 1st/3rd trimester samples for Gipuzkoa. *P gt; 0.05 and **P gt; 0.001 where P values were calculated using the χ2 test (categorical variables) or Mann–Whitney test (continuous) between Sabadell women (3rd trimester sub-cohort) and Gipuzkoa women. (DOCX 68 kb)
Additional file 8: Table S3. Decomposition of variance in Birthweight. (DOCX 41 kb)
Additional file 9: Figure S5. Potential sources of variation in metabolite signatures of fetal growth in 1st trimester from epidemiological data such as lifestyle and clinical parameters in Sabadell. (PDF 93 kb)
