# Supplementary material for: Maternal urinary metabolic signatures of fetal growth and associated clinical and environmental factors in the INMA study
Source: BMC Med. 2016 Nov 4;14:177. doi: 10.1186/s12916-016-0706-3 (PMC5097405; doi:10.1186/s12916-016-0706-3)
Supplement: Additional file 1: — Supplementary methods. (DOCX 38 kb) [file 12916_2016_706_MOESM1_ESM.docx]

## Additional file 1

## Supporting Information Methods

### Fetal growth assessment

Ultrasound examinations were routinely performed at gestational wks 12, 20, and 34 by specialised obstetricians and anthropometric measures were obtained by trained midwives at delivery. Fetal growth was assessed in this study based on continuous body weight at four time points during pregnancy (12^th^, 34^th^ wk and at birth). Fetal weight (FW) was estimated based on ultrasounds measures of abdominal circumference, biparietal diameter and femur length, using the Hadlock algorithm. Fetal growth scores or standard deviation scores (z-scores) were obtained using longitudinal growth curves calculated for each individual adjusting for constitutional factors known to affect fetal growth (i.e. maternal age, height, parity, pre-pregnancy weight, country of origin, father's height and fetal sex). Two types of z-scores are presented: unconditional z-scores at a certain time point which describe the size of a fetus at this time and conditional z-score which describe the growth of a fetus during the respective time interval, i.e. 12^th^-20^th^, 20^th^-34^th^ and 12^th^-34^th^ wks of gestation (10). Measures at birth included body weight and placental weight and were scaled to z-scores by subtracting the mean and dividing by the standard deviation. The advantage of converting measurements into z-scores is that it eliminates variability by gestational age in the case of fetal growth measurements, allowing measurements to be compared. A positive z-score indicates a larger and a negative value a smaller than expected body weight.

## Covariates

Gestational age was calculated from the date of the last menstrual period (LMP) which was self-reported and was confirmed using ultrasound examination in 12 wk of gestation. When the date did not match the ultrasound measurement estimation by 7 days or more, the gestational age was corrected using its relationship to the crown-rump length.

## Metabonomic protocol

### Data acquisition

Urine samples were thawed, vortexed, and allowed to stand for 10 min prior to mixing aliquots (400 μL) with phosphate buffer [200 μL, 0.2 M containing 99% deuterium oxide (D_2_O), 1 mM 3-(trimethylsilyl)-[2,2,3,3-^2^H_4_]-propionic acid sodium salt (TSP), and 3 mM sodium azide] and centrifuged at 15900 x g for 10 min. Supernatants (550 μL) were transferred into NMR tubes (507-HP-7, Norell, Landisville, NJ). The D_2_O provided a field frequency lock, and TSP provided a chemical shift reference (^1^H, δ 0). ^1^H NMR spectra were acquired on a Bruker Avance spectrometer (Bruker Biospin, Rheinstetten, Germany) operating at a ^1^H frequency of 600.13 MHz and a temperature of 300 K, using a Bruker TXI probe and an automated sample handling carousel (Bruker). NMR spectra were acquired using the standard one-dimensional solvent suppression pulse sequence (relaxation delay, 90° pulse, 4 μs delay, 90° pulse, mixing time, 90° pulse, acquire FID). For each sample, 128 transients were collected into 64K data points using a spectral width of 12000 Hz with a relaxation delay of 4 s, a mixing time of 100 ms, and an acquisition time of 2.73 s. A line-broadening function of 0.3 Hz was applied to all spectra prior to Fourier transformation (FT) (Beckonert et al., 2007). Pooled urine samples from the Sabadell cohort were used as quality control (QC) samples to monitor analytical stability and sample degradation over the NMR run.

### Data processing

After manual phasing in Topspin, all NMR spectra (spectral region δ 10 – 0.5) were imported into MATLAB 7.3.1 (MathWorks) and were referenced and corrected for baseline distortion using an in-house script developed by Dr. Rachel Cavill, Dr. Hector Keun and Dr. Tim Ebbels at Imperial College London, UK. The spectral region δ 4.0-6.1 containing residual water and urea resonances were removed prior to probabilistic quotient normalisation (Dieterle et al., 2006). Integrals of single representative peaks were chosen on the basis of being present in a high proportion of spectra, having a high signal-to-noise ratio, and exhibiting limited overlap with other peaks.  Certain metabolites with a low signal-to-noise ratio (specifically 0.58 (s), 0.63 (s), 0.92(s) and 0.93(s)) were quantified using a Matlab peak fitting program for time-series signals, which uses an unconstrained [non-linear optimization algorithm](http://terpconnect.umd.edu/~toh/spectrum/CurveFittingC.html) to decompose a complex signal into its component parts (O'Haver).

### Metabolite annotation

Discriminatory metabolites were structurally annotated to specific metabolites using published studies (Yap et al., 2010, Salek et al., 2007), online databases (HMDB) (Wishart et al., 2009) and/or confirmed by 2D NMR experiments on a selected sample including homonuclear ^1^H-^1^H correlation spectroscopy (COSY), ^1^H-^1^H total correlation spectroscopy (TOCSY) and ^1^H-^13^C NMR heteronuclear spectroscopy. Further standard chemical spike-in experiments were required for certain final metabolite annotations. Additionally, to further annotate NMR signals in particular in overlapped regions, HPLC-off line fractionation was used (details in next paragraph) and 800MHz ^1^H NMR spectra were acquired on each of the fractions. 0.63 ppm (s) and 0.92 ppm (s) signals assignment to 5β-Pregnane-3α,20α-diol-3α-glucuronide was confirmed after analysing a purified fraction by LC-MS/MS in positive mode on a Xevo G2-S (Waters) and matching parent and fragment masses to a public library (Metlin) and to the chemical standard spiked into the original samples. According to our LC-MS/MS data on selected fractions of interest, spiked-in NMR experiments and literature, a mixture of pregnanolone glucuronide isomers is likely to contribute to the signal arising at 0.56 (s). Oestrogen metabolites are likely to contribute to the signals arising at 0.78(s) according to spike-in experiments and a previous study but was not fully annotated due to its low abundance in the original samples (28).

### HPLC fractionation protocol

A pooled urine sample from the Sabadell samples was fractionated by repeated separation using a Waters Acquity UPLC system equipped with a Waters Fraction Collector III. The urine sample was injected 33 times using a full loop injection of 20 µL and the chromatographic separation reproducibility was monitored using a photodiode array detector (PDA) scanning wavelengths from 210 to 500nm. A reversed-phase separation was performed using a 4.6 x 150mm Waters Atlantis T3 column with 3µm particle size held at 25°C for the duration of the experiment. The separation was performed using 0.1% (v/v) formic acid in water (A) and 0.1% (v/v) formic acid in methanol (B). At the time of injection, the LC system was held at initial conditions (100% A) for one minute, followed by a 15 minute linear gradient to 5:95 A:B. Four minutes of cleaning were performed at final conditions followed by a one minute linear return to initial conditions and four minutes of equilibration prior to the next injection (30 min total cycle time). The solvent flow rate was held constant at 1mL/min. Effluent from the LC system was collected into 120 fractions (9 seconds each) following a 1.5 minute hold to clear the system volume.

The 120 fractions of accumulated material from the repeated separations were evaporated to dryness using a TurboVap® LV (Biotage) supplied with nitrogen and 37°C heat. The residue of each fraction was dissolved in deuterated water.
