# Supplementary material for: Maternal urinary metabolic signatures of fetal growth and associated clinical and environmental factors in the INMA study
Source: BMC Med. 2016 Nov 4;14:177. doi: 10.1186/s12916-016-0706-3 (PMC5097405; doi:10.1186/s12916-016-0706-3)
Supplement: Additional file 2: Table S1. — List of metabolite integrals and assignments obtained from NMR urine spectra. Abbreviations: UA, unassigned. *The method of multiple spike-in of authentic standards in the original sample allowed to validate the identity of NMR signals. The identity of P3G was confirmed by chromatographic isolation of the target feature (SI Methods) and comparison of MS/MS spectra to an authentic reference compound in accordance with reported guidelines for metabolite identification (Sumner et al. 2014). **Citation for STORM: Subset Optimization by Reference Matching (STORM): An Optimized Statistical Approach for Recovery of Metabolic Biomarker Structural Information from 1H NMR Spectra of Biofluids. Joram M. Posma, Isabel Garcia-Perez, Maria De Iorio, John C. Lindon, Paul Elliott, Elaine Holmes, Timothy M. D. Ebbels, and Jeremy K. Nicholson. Analytical Chemistry 2012 84 (24), 10694-10701 DOI: 10.1021/ac302360v. (DOCX 43 kb) [file 12916_2016_706_MOESM2_ESM.docx]

### Additional file 2: Table S1

**List of metabolite integrals and assignments obtained from NMR urine spectra**

|  | Metabolite ID | Abbreviation | Chemical shift | Integral range 1 (ppm) | Integral range 2 (ppm) | Assignment | Metabolic class |
| --- | --- | --- | --- | --- | --- | --- | --- |
| 1 | Pregnanolone-3G | Pn3-G | 0.58 (s) | 0.55 | 0.57 | Fraction, spike-in* | Steroid and steroid derivates |
| 2 | Pregnanediol-3G | P3-G | 0.63 (s) | 0.6176 | 0.64 | Fraction, spike-in, LC-MS/MS | Steroid and steroid derivates |
| 3 | UA 0.75(d) | - | 0.75 (d) | 0.741 | 0.76 | - | Steroid and steroid derivates |
| 4 | Estrogen metabolite | - | 0.78 (s) | 0.7774 | 0.79 | Fraction, Spike-in | Steroid and steroid derivates |
| 5 | UA 0.93(s) | - | 0.93 (s) | 0.9311 | 0.94 | - | Unknown |
| 6 | Leucine | Leu | 0.96 (t) | 0.9503 | 0.97 | 2D NMR | BCAAs catabolism |
| 7 | Isoleucine | Iso | 1.01 (d) | 1.005 | 1.03 | 2D NMR | BCAAs catabolism |
| 8 | Valine | Val | 1.05 (d) | 1.034 | 1.06 | 2D NMR | BCAAs catabolism |
| 9 | 3-hydroxyisobutyrate | 3-HB | 1.08 (d) | 1.063 | 1.09 | 2D NMR, fractions, spike-in | BCAAs catabolism |
| 10 | 4-deoxyerythronic acid | 4-DEA | 1.11 (d) | 1.097 | 1.12 | 2D NMR | Glucose metabolism |
| 11 | UA 1.15 (d) [3.45, 3.54] | - | 1.15 (d) | 1.132 | 1.16 | 2D NMR (organic acid with similar structure to 3-hydroxyisobutyrate) | Glucose metabolism |
| 12 | 3-hydroxybutyrate/3-aminoisobutyrate | 3-HB/3-AB | 1.20 (ov.) | 1.17 | 1.21 | 2D NMR | Glucose metabolism |
| 13 | 4-deoxythreonic acid | 4-DTA | 1.24 (d) | 1.222 | 1.24 | 2D NMR | Glucose metabolism |
| 14 | UA 1.25 | - | 1.25 (d) | 1.244 | 1.26 | - | Unknown |
| 15 | 3-hydroxyisovalerate | 3-HV | 1.27 (s) | 1.264 | 1.28 | 2D NMR | Glucose metabolism |
| 16 | UA 1.29(s) | - |  | 1.282 | 1.29 | - | Unknown |
| 17 | Lactate | Lac | 1.33 (d) | 1.32 | 1.35 | 2D NMR | Glucose metabolism |
| 18 | 2-hydroxyisobutyrate | 2-HB | 1.36 (s) | 1.355 | 1.37 | 2D NMR, spike-in | BCAAs catabolism |
| 19 | Alanine | Ala | 1.48 (d) | 1.472 | 1.51 | 2D NMR | Glucose metabolism |
| 20 | UA 1.67(s) | - | 1.67 (s) | 1.655 | 1.68 | - | Unknown |
| 21 | Lysine | Lys | 1.73 (m) | 1.701 | 1.76 | 2D NMR, Chenomx | Glucose metabolism |
| 22 | Acetate | - | 1.92 (s) | 1.914 | 1.93 | Literature | TCA cycle |
| 23 | UA 1.99(t) | - | 1.99(t) | 1.97 | 2.00 | - | Unknown |
| 24 | *N-*acetyl glycoprotein fragments | NAG | 2.04 (s) | 2.022 | 2.05 | Literature | Amino sugar and nucleotide sugar metabolism |
| 25 | N-acetyl neuraminic acid | NAC | 2.06 (s) | 2.054 | 2.09 | Literature | Amino sugar and nucleotide sugar metabolism |
| 26 | Acetone | - | 2.24 (s) | 2.229 | 2.25 | Literature | Oxidative stress |
| 27 | P-cresol sulfate | PCS | 2.35 (s) | 2.339 | 2.355 | 2D NMR | Bacterial origin |
| 28 | Pyroglutamate (5-oxoproline) | 5-OP | 2.40 (m) | 2.387 | 2.40 | 2D NMR, fractions, spike-in | Oxidative stress |
| 29 | Succinate | - | 2.41 (s) | 2.4 | 2.42 | Literature | TCA cycle and mitochondrial oxidative stress |
| 30 | Glutamine | Glu | 2.45 (m) | 2.43 | 2.47 | 2D NMR | Glucose metabolism |
| 31 | Citrate | - | 2.55 (d) | 2.5 | 2.58 | 2D NMR | TCA cycle and mitochondrial oxidative stress |
| 32 | 3-aminoisobutyrate | 3-AB | 2.62 (m) | 2.587 | 2.64 | 2D NMR | BCAAs catabolism |
| 33 | Dimethylamine | DMA | 2.72 (s) | 2.716 | 2.74 | Literature | Alkylamines |
| 34 | UA 2.78(s) | - | 2.78 (s) | 2.774 | 2.79 | - | Unknown |
| 35 | UA 2.83(s) | - | 2.83 (s) | 2.818 | 2.85 | - | Unknown |
| 36 | Trimethylamine | TMA | 2.87 (s) | 2.861 | 2.88 | Literature | Alkylamines/bacterial origin |
| 37 | UA 2.9 (N-acetyl peak) | - | 2.90 | 2.9 | 2.91 | 2D NMR, spike-in | Oxidative stress |
| 38 | Dimethylglycine | DMG | 2.93 (s) | 2.923 | 2.94 | Spike-in | Unknown |
| 39 | Creatinine+creatine | Creat2 | 3.05 (s) ov. | 3.027 | 3.07 | Literature | Amino Acids and Derivatives |
| 40 | Choline | - | 3.20 (s) | 3.201 | 3.21 | Literature | Gluconeogenesis |
| 41 | Carnitine | - | 3.23 (s) | 3.223 | 3.24 | Literature | Oxidative stress |
| 42 | Trimethylamine oxide | TMAO | 3.27 (s) | 3.261 | 3.29 | Literature | Alkylamines/bacterial origin |
| 43 | Proline betaine | PB | 3.30 (s) | 3.297 | 3.31 | Literature | Pyrrolidines |
| 44 | Scyllo-inositol | SIN | 3.36 (s) | 3.362 | 3.37 | Literature | Nicotinate and nicotinamide metabolism |
| 45 | Glycine | Gly | 3.57 (s) | 3.561 | 3.58 | Literature | Gluconeogenesis |
| 46 | UA 3.8(s) | - | 3.8(s) | 3.792 | 3.81 | - | unknown |
| 47 | UA 3.93(s) | - | 3.93(s) | 3.927 | 3.94 | - | Unknown |
| 48 | UA 3.95(s) | - | 3.95(s) | 3.946 | 3.96 | - | Unknown |
| 49 | UA 6.48(d) | - | 6.48(d) | 6.477 | 6.49 | - | Unknown |
| 50 | Fumarate | - | 6.52 (s) | 6.521 | 6.53 | Literature | Oxidative stress |
| 51 | UA 6.59(s) | - | 6.59 (s) | 6.585 | 6.61 | - | Unknown |
| 52 | Furoylglycine | FG | 6.64 (dd) | 6.636 | 6.66 | 2D NMR, fractions | Nicotinate and nicotinamide metabolism |
| 53 | *N-*methyl-2-pyridone-5-carboxamide | 2PY | 6.66 (s) | 6.657 | 6.67 | 2D NMR | Nicotinate and nicotinamide metabolism |
| 54 | p-hydroxyphenylacetate | HPA | 6.87 (d) | 6.851 | 6.88 | 2D NMR, STORM**, spike-in | Bacterial origin |
| 55 | Tyrosine | Tyr | 6.90 (d) | 6.89 | 6.92 | 2D NMR | Amino Acids and Derivatives |
| 56 | UA 7.32 | - | 7.32 | 7.306 | 7.34 | - | Unknown |
| 57 | Phenylacetylglutamine | PAG | 7.37 (t) | 7.343 | 7.38 | 2D NMR | Bacterial origin |
| 58 | 3-Indoxylsulfate | 3-IS | 7.51 (d) | 7.494 | 7.52 | 2D NMR, spike-in | Bacterial origin |
| 59 | Hippurate | - | 7.55 (t) | 7.527 | 7.58 | 2D NMR | Bacterial origin |
| 60 | UA 7.68(s) | - | 7.68(s) | 7.672 | 7.69 | - | Unknown |
| 61 | Formate | - | 8.46 (s) | 8.453 | 8.47 | Literature | Organic acids |
| 62 | UA 8.79(d) | - | 8.79(d) | 8.772 | 8.81 | - | Nicotinate and nicotinamide metabolism |
| 63 | *N-*Methylnicotinic acid | NMNA | 9.13 (s) | 9.113 | 9.14 | 2D NMR, spike-in | Nicotinate and nicotinamide metabolism |
| 64 | *N*¹-Methyl-nicotinamide | NMN | 9.28 (s) | 9.269 | 9.30 | 2D NMR, spike-in | Nicotinate and nicotinamide metabolism |

Abbreviations: UA, unassigned

* The method of multiple spike-in of authentic standards in the original sample allowed to validate the identity of NMR signals. The identity of P3G was confirmed by chromatographic isolation of the target feature (SI Methods) and comparison of MS/MS spectra to an authentic reference compound in accordance with reported guidelines for metabolite identification (14).

**Citation for STORM: Subset Optimization by Reference Matching (STORM): An Optimized Statistical Approach for Recovery of Metabolic Biomarker Structural Information from 1H NMR Spectra of Biofluids. Joram M. Posma, Isabel Garcia-Perez, Maria De Iorio, John C. Lindon, Paul Elliott, Elaine Holmes, Timothy M. D. Ebbels, and Jeremy K. Nicholson. *Analytical Chemistry* 2012 84 (24), 10694-10701 DOI: 10.1021/ac302360v
