# Supplementary figures and images for: Maternal urinary metabolic signatures of fetal growth and associated clinical and environmental factors in the INMA study
Source: BMC Med. 2016 Nov 4;14:177. doi: 10.1186/s12916-016-0706-3 (PMC5097405; doi:10.1186/s12916-016-0706-3)

# 1st Sabadell

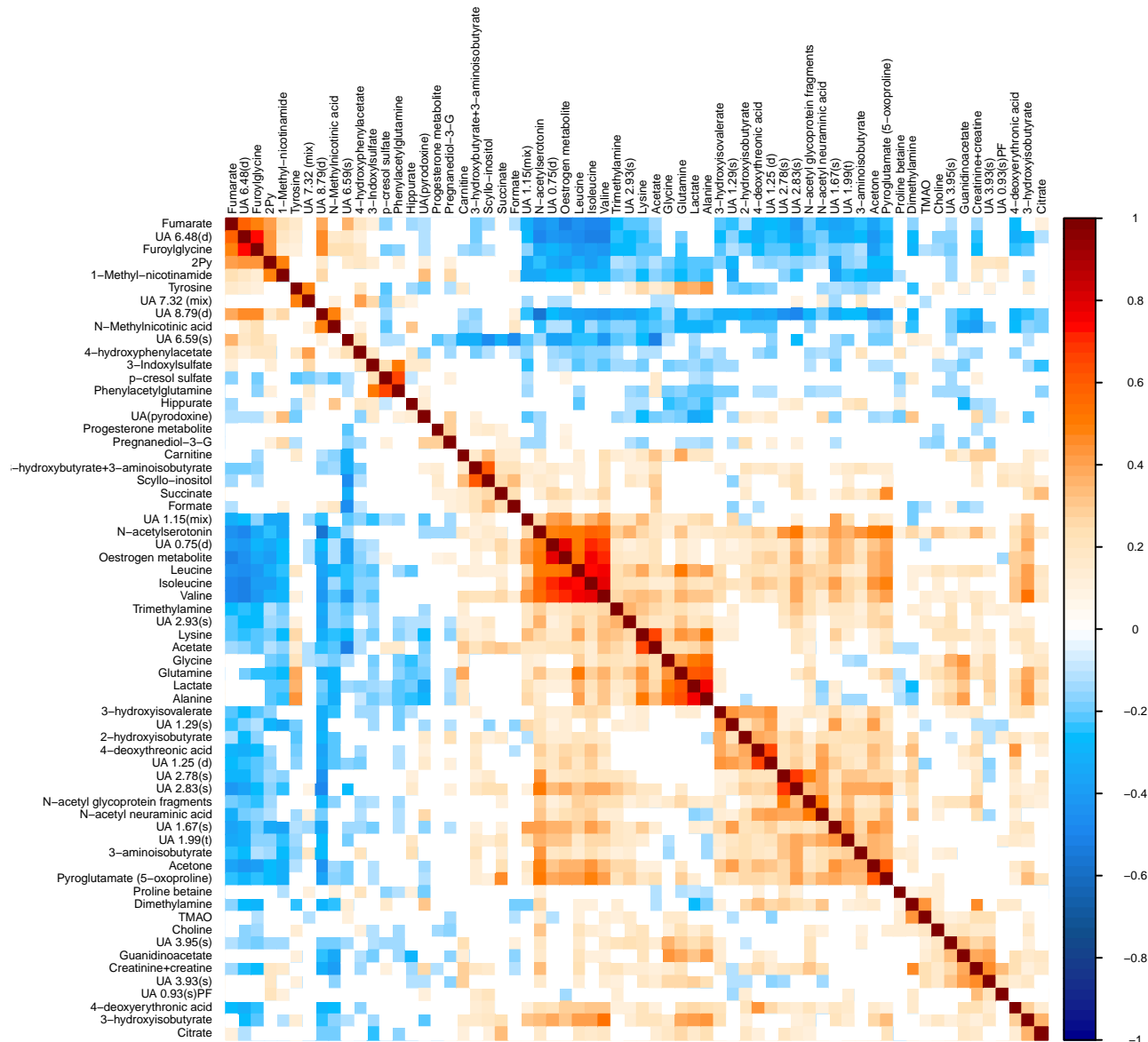

Supplement: Additional file 3: Figure S1. — Heatmap of metabolite inter-correlation in urinary 1H NMR spectral profiles from Gipuzkoa at week 12 of gestation (metabolite order created using the complete linkage method for hierarchical clustering). (PDF 48 kb) [file 12916_2016_706_MOESM3_ESM.pdf]

# 3rd Sabadell

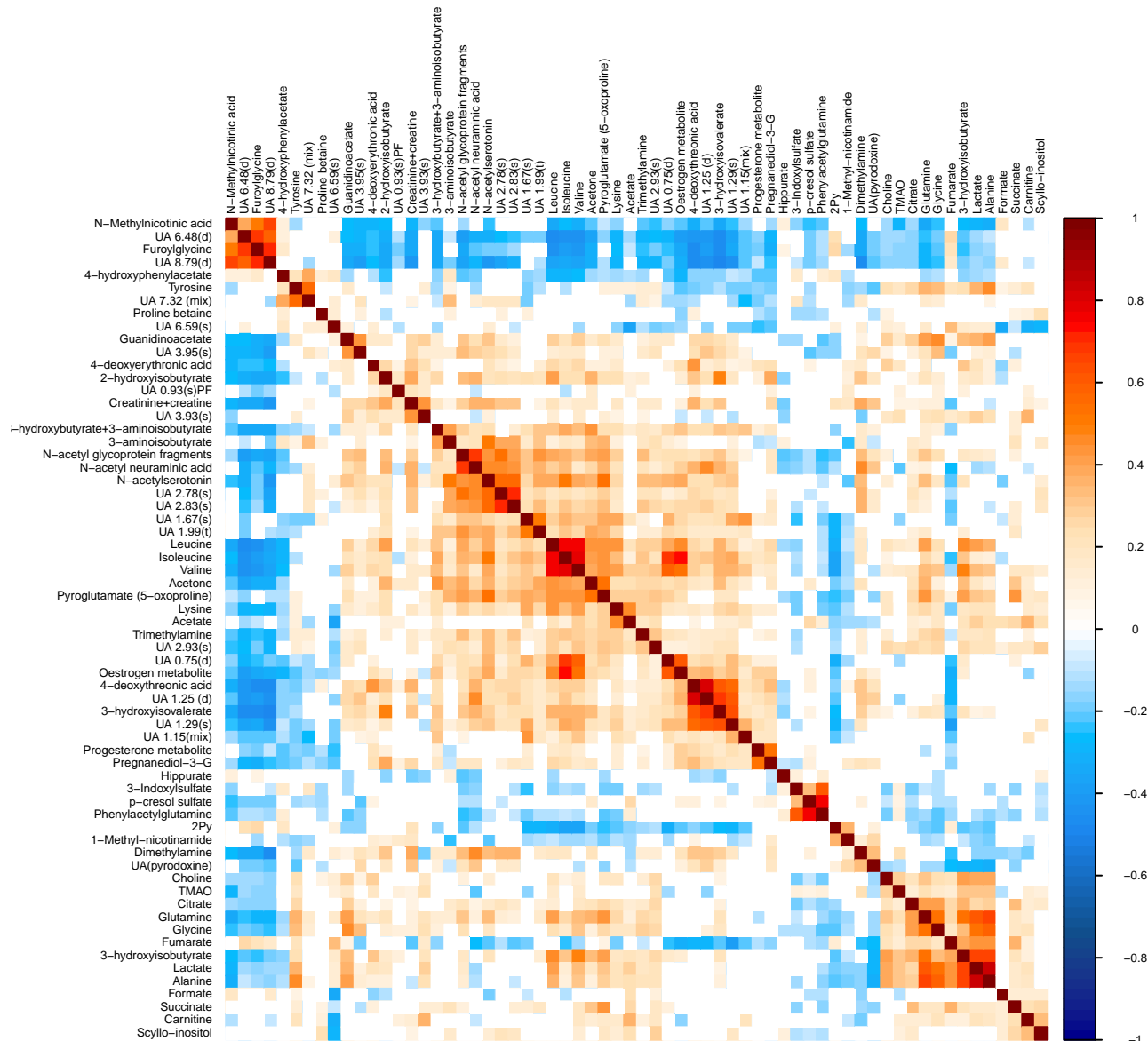

Supplement: Additional file 4: Figure S2. — Heatmap of metabolite inter-correlation in urinary 1H NMR spectral profiles from Gipuzkoa at week 34 of gestation (metabolite order created using the complete linkage method for hierarchical clustering). (PDF 47 kb) [file 12916_2016_706_MOESM4_ESM.pdf]

# 1st Gipuzkoa

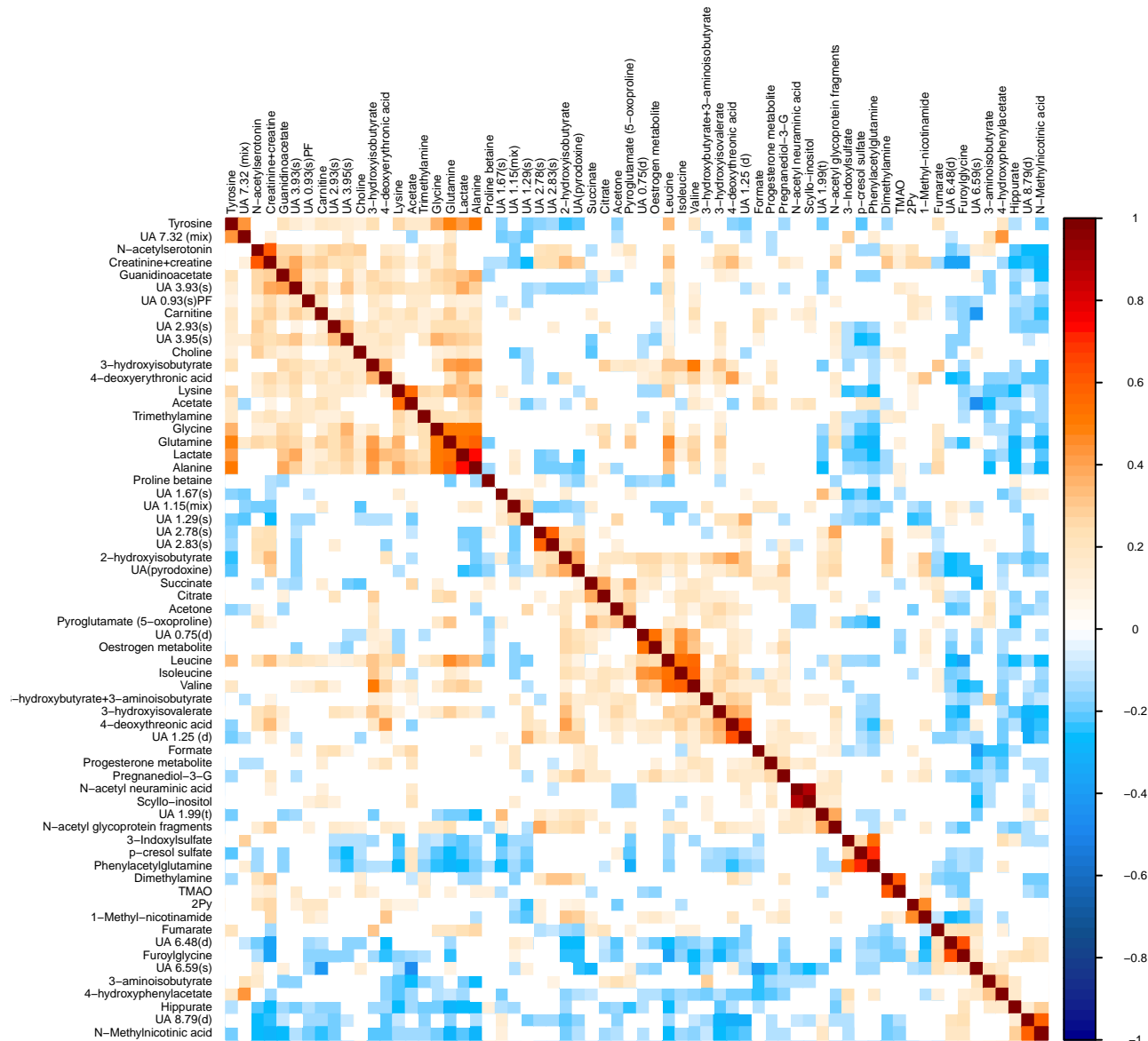

Supplement: Additional file 5: Figure S3. — Heatmap of metabolite inter-correlation in urinary 1H NMR spectral profiles from Sabadell at week 12 of gestation (metabolite order created using the complete linkage method for hierarchical clustering). (PDF 48 kb) [file 12916_2016_706_MOESM5_ESM.pdf]

### 3rd Gipuzkoa

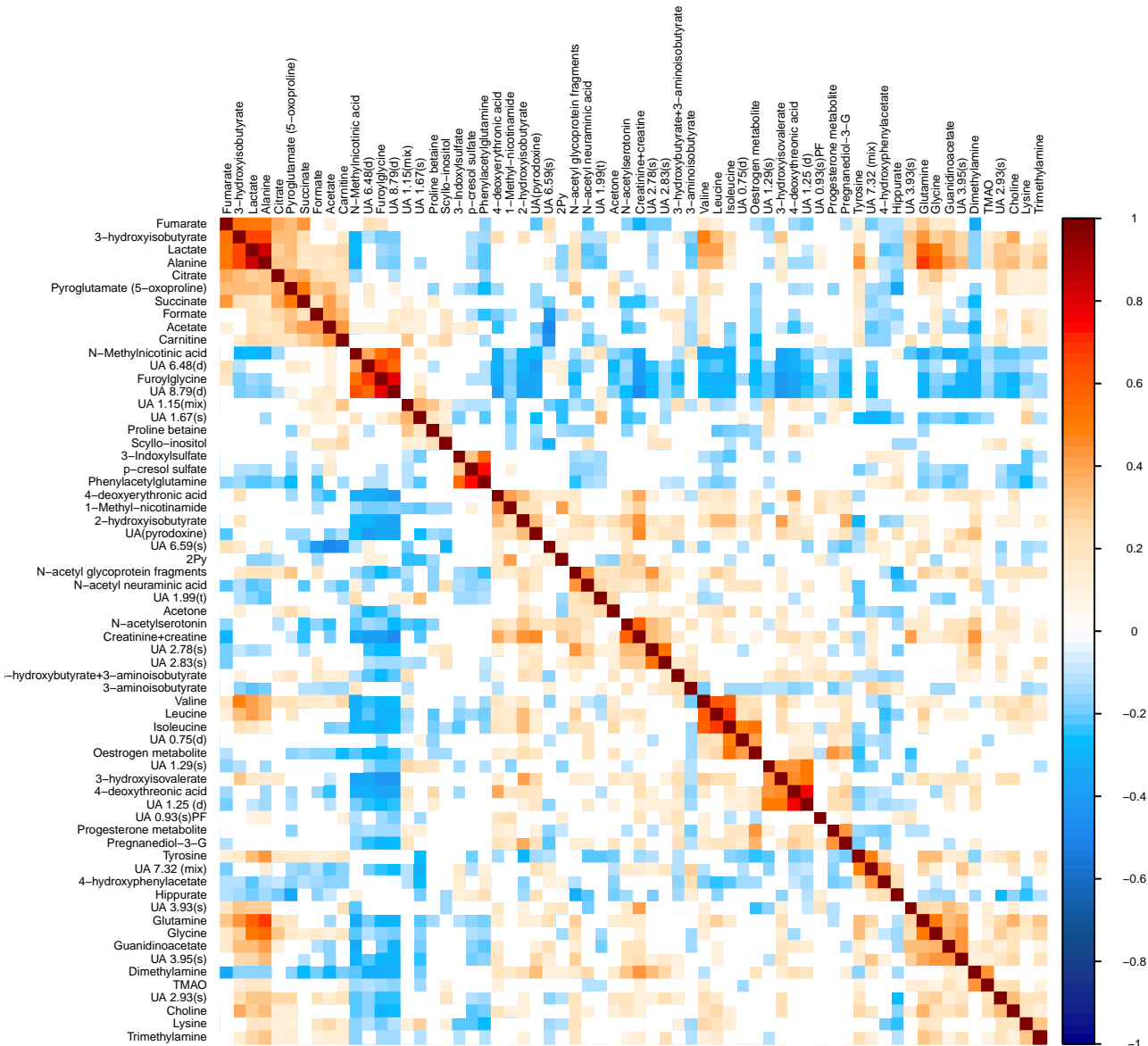

Supplement: Additional file 6: Figure S4. — Heatmap of metabolite inter-correlation in urinary 1H NMR spectral profiles from Sabadell at week 34 of gestation (metabolite order created using the complete linkage method for hierarchical clustering). (PDF 48 kb) [file 12916_2016_706_MOESM6_ESM.pdf]
