# Supplementary material for: Maternal urinary metabolic signatures of fetal growth and associated clinical and environmental factors in the INMA study
Source: BMC Med. 2016 Nov 4;14:177. doi: 10.1186/s12916-016-0706-3 (PMC5097405; doi:10.1186/s12916-016-0706-3)
Supplement: Additional file 7: Table S2. — Characteristics of women in Gipuzkoa and Sabadell during pregnancy included in theanalysis. Since the population characteristics in the Gipuzkoa cohort are almost identical between the first and third trimesters (only 5 women are different in the third trimester), the table is combined for 1st/3rd trimester samples for Gipuzkoa. *P gt; 0.05 and **P gt; 0.001 where P values were calculated using the χ2 test (categorical variables) or Mann–Whitney test (continuous) between Sabadell women (3rd trimester sub-cohort) and Gipuzkoa women. (DOCX 68 kb) [file 12916_2016_706_MOESM7_ESM.docx]

## Additional file 7: Table S2

## Maternal characteristics in the study population

Since the population characteristics in the Gipuzkoa cohort are almost identical between the first and third trimesters (only 5 women are different in the third trimester), the table is combined for 1st/3rd trimester samples for Gipuzkoa. *P < 0.05 and **P < 0.001 where P values were calculated using the χ2 test (categorical variables) or Mann–Whitney test (continuous) between the two cohorts for the women included in 3rd trimester.

Table: characteristics of women in Gipuzkoa and Sabadell during pregnancy included in the analysis (different sub-cohorts exist for women who gave samples in 1st trimester or in 3rd trimester). P values were calculated using the Chi-squared test (categorical variables) or Mann-Whitney test (continuous) between Sabadell women (3rd trimester sub-cohort) and Gipuzkoa women.

|  |  |  | **Gipuzkoa* - Included in the analysis (combined weeks 12 and 34, n=419)** | | **Sabadell - Included in the analysis (1st trimester, n=394)** | | **Sabadell - Included in the analysis (3rd trimester, n=469)** | |  |
| --- | --- | --- | --- | --- | --- | --- | --- | --- | --- |
| **Category** | **Parameter** | **Details** | **Median/**  **Count** | **Interquartile**  **range/%** | **Median/Count** | **Interquartile**  **range/%** | **Median/**  **Count** | **Interquartile**  **range/%** | **p-value for cohort comparison** |
| **Socio-demographic** | Mother age |  | 31 | 29 - 33 | 30 | 28 - 33 | 30 | 28 - 33 | * |
|  | Missing |  | 0 | 0 | 0 | 0 | 1 | 0 |  |
|  | Mother ethnicity | Spain | 369 | 89.6 | 342 | 86.8 | 398 | 84.9 | ** |
|  |  | Latin America | 8 | 1.9 | 29 | 7.4 | 36 | 7.7 |  |
|  |  | Others | 4 | 1.0 | 14 | 3.6 | 13 | 2.8 |  |
|  | Missing |  | 31 | 7.5 | 9 | 2.3 | 22 | 4.7 |  |
|  | Country of birth | Spanish | 398 | 96.6 | 346 | 87.8 | 411 | 87.6 | ** |
|  |  | Foreign | 14 | 3.4 | 43 | 10.9 | 51 | 10.9 |  |
|  | Missing |  | 0 | 0.0 | 5 | 1.3 | 7 | 1.5 |  |
|  | Maternal social class | CS I+II | 130 | 31.6 | 84 | 21.3 | 91 | 19.4 | ** |
|  |  | CS III | 124 | 30.1 | 116 | 29.4 | 135 | 28.8 |  |
|  |  | CS IV+V | 158 | 38.3 | 194 | 49.2 | 243 | 51.8 |  |
|  | Missing | Others | 0 | 0.0 | 0 | 0.0 | 0 | 0.0 |  |
|  | Paternal social class | CSP I+II | 109 | 26.5 | 81 | 20.6 | 91 | 19.4 |  |
|  |  | CSP III | 56 | 13.6 | 62 | 15.7 | 72 | 15.4 |  |
|  |  | CSP IV+V | 242 | 58.7 | 225 | 57.1 | 274 | 58.4 |  |
|  | Missing |  | 5 | 1.2 | 26 | 6.6 | 32 | 6.8 |  |
|  | Maternal education level | Primary or without education | 48 | 11.6 | 108 | 27.5 | 137 | 29.2 | ** |
|  |  | Secondary | 142 | 34.5 | 160 | 40.6 | 195 | 41.6 |  |
|  |  | University | 221 | 53.6 | 123 | 31.2 | 135 | 28.8 |  |
|  | Missing |  | 1 | 0.2 | 3 | 0.8 | 2 | 0.4 |  |
|  | Paternal education | Primary | 87 | 21.1 | 141 | 35.8 | 166 | 35.4 |  |
|  |  | Not able to write and read | 7 | 1.7 | 1 | 0.3 | 0 | 0.0 | ** |
|  |  | Without education or unfinished primary school | 8 | 1.9 | 7 | 1.8 | 9 | 1.9 |  |
|  |  | Secondary | 196 | 47.6 | 163 | 41.4 | 197 | 42.0 |  |
|  |  | University | 109 | 26.5 | 78 | 19.8 | 92 | 19.6 |  |
|  | Missing |  | 5 | 1.2 | 4 | 1.0 | 5 | 1.1 |  |
| **Lifestyle** | Hours of sleep in 1st trimester |  | 8 | 8-9 | 8 | 7-9 | 8 | 7-9 |  |
|  | Missing |  | 12 |  | 1 |  | 1 |  |  |
|  | Overall physical activity in 1st trimester | Moderate activity | 161 | 39.1 | 181 | 45.9 | 210 | 44.8 |  |
|  |  | Little activity | 101 | 24.5 | 111 | 28.2 | 131 | 27.9 |  |
|  |  | Intense or sufficient activity | 132 | 32.0 | 100 | 25.4 | 125 | 26.7 |  |
|  | Missing |  | 18 | 4.3 | 2 | 0.5 | 3 | 0.6 |  |
|  | Hours of sleep in 3rd trimester |  | 8 | 7-8 | 8 | 7-8 | 8 | 7-8 |  |
|  | Missing |  | 3 |  | 0 |  | 1 |  | ** |
|  | Overall physical activity in 3rd trimester | Moderate activity | 203 | 49.3 | 138 | 35.0 | 166 | 35.4 |  |
|  |  | Little activity | 125 | 30.3 | 178 | 45.2 | 213 | 45.4 | ** |
|  |  | Intense or sufficient activity | 81 | 19.7 | 78 | 19.8 | 89 | 19.0 |  |
|  | Missing |  | 3 | 0.7 | 0 |  | 1 | 0.2 |  |
|  | Night work | Day shift | 338 | 82.0 | 328 | 83.2 | 386 | 82.3 |  |
|  |  | Night shift | 19 | 4.6 | 20 | 5.1 | 20 | 4.3 |  |
|  | Missing |  | 55 | 13.3 | 46 | 11.7 | 63 | 13.4 |  |
|  | Zone of residence | Rural | 51 | 12.4 | 0 | 0.0 | 0 | 0.0 |  |
|  |  | Suburban | 163 | 39.6 | 0 | 0.0 | 0 | 0.0 | ** |
|  |  | Urban | 198 | 48.1 | 393 | 99.7 | 468 | 99.8 |  |
|  | Missing |  | 0 |  | 1 | 0.3 | 1 | 0.2 |  |
|  | Total passive smoking | No exposition | 174 | 42.2 | 142 | 36.0 | 174 | 37.1 |  |
|  |  | At least 1 between home/work/rest/leisure | 180 | 43.7 | 167 | 42.4 | 193 | 41.2 | * |
|  |  | At least 2 between home/work/rest/leisure | 51 | 12.4 | 73 | 18.5 | 85 | 18.1 |  |
|  |  | At least 3 between home/work/rest/leisure | 6 | 1.5 | 12 | 3.0 | 15 | 3.2 |  |
|  | Missing |  | 1 | 0.2 | 0 |  | 2 | 0.4 |  |
|  | Average daily intake of alcohol (g) | 0.04-1g/day | 158 | 38.3 | 124 | 31.5 | 147 | 31.3 |  |
|  |  | 1g/day | 25 | 6.1 | 38 | 9.6 | 40 | 8.5 |  |
|  |  | None | 226 | 54.9 | 232 | 58.9 | 281 | 59.9 |  |
|  | Missing |  | 3 | 0.7 | 0 |  | 1 | 0.2 |  |
|  | Smoking before pregnancy | between 1 and 10 cig/day | 122 | 29.6 | 101 | 25.6 | 121 | 25.8 |  |
|  |  | more than 10 cig/day | 80 | 19.4 | 93 | 23.6 | 110 | 23.5 |  |
|  |  | No smoking | 207 | 50.2 | 197 | 50.0 | 233 | 49.7 |  |
|  | Missing |  | 3 | 0.7 | 3 | 0.8 | 5 | 1.1 |  |
|  | Smoking at the beginning of pregnancy | between 1 and 10 cig/day | 52 | 12.6 | 53 | 13.5 | 64 | 13.6 |  |
|  |  | more than 10 cig/day | 36 | 8.7 | 64 | 16.2 | 73 | 15.6 | * |
|  |  | No smoking | 323 | 78.4 | 276 | 70.1 | 330 | 70.4 |  |
|  | Missing |  | 1 | 0.2 | 1 | 0.3 | 2 | 0.4 |  |
|  | Smoking in 1st trimester | between 1 and 10 cig/day | 39 | 9.5 | 53 | 13.5 | 63 | 13.4 |  |
|  |  | more than 10 cig/day | 2 | 0.5 | 7 | 1.8 | 7 | 1.5 |  |
|  |  | No smoking | 370 | 89.8 | 334 | 84.8 | 398 | 84.9 |  |
|  | Missing |  | 1 | 0.2 | 0 |  | 1 | 0.2 |  |
|  | BMI before pregnancy | <18.5 (Underweight) | 13 | 3.2 | 24 | 6.1 | 27 | 5.8 |  |
|  |  | 18.5-25 (Healthy) | 314 | 76.2 | 268 | 68.0 | 310 | 66.1 | * |
|  |  | 25.1-30 (Overweight) | 64 | 15.5 | 72 | 18.3 | 93 | 19.8 |  |
|  |  | >30 (Obese) | 21 | 5.1 | 30 | 7.6 | 39 | 8.3 |  |
|  | Missing |  | 0 |  | 0 |  | 0 |  |  |
| **Diet** | coffee/infusions in 1st trimester(g) |  | 200 | 57 - 314 | 185.8 | 13 - 200 | 185.8 | 13 - 200 |  |
|  | Missing |  | 11 |  | 1 |  | 1 |  | ** |
|  | intake of calories in 1st trimester(Kcal) |  | 1851 | 1601 - 2107 | 2075 | 1785 - 2410 | 2075 | 1782 - 2412 |  |
|  | Missing |  | 11 |  | 1 |  | 1 |  | ** |
|  | total fat in 1st trimester(g) |  | 71.63 | 57 - 85 | 94.05 | 77 - 109 | 94.05 | 77 - 110 |  |
|  | Missing |  | 11 |  | 1 |  | 1 |  | ** |
|  | consumption of dairy  in 1st trimester(g) |  | 367.9 | 282 - 589 | 356.7 | 254 - 596 | 356.7 | 248 - 604 |  |
|  | Missing |  | 11 |  | 1 |  | 1 |  |  |
|  | consumption of eggs  in 1st trimester(g) |  | 21.62 | 22 - 22 | 21.62 | 22 - 22 | 21.62 | 22 - 22 |  |
|  | Missing |  | 11 |  | 1 |  | 1 |  | * |
|  | meat  in 1st trimester(g) |  | 91.36 | 66 - 115 | 127.6 | 102 - 156 | 127.6 | 101 - 157 |  |
|  | Missing |  | 11 |  | 1 |  | 1 |  | ** |
|  | fish in 1st trimester(g) |  | 67.28 | 47 - 88 | 65.49 | 43 - 92 | 65.49 | 43 - 92 |  |
|  | Missing |  | 11 |  | 1 |  | 1 |  |  |
|  | vegetables in 1st trimester(g) |  | 192.2 | 145 - 257 | 213.1 | 148 - 282 | 213.1 | 150 - 292 |  |
|  | Missing |  | 11 |  | 1 |  | 1 |  | * |
|  | fruits in 1st trimester(g) |  | 309.7 | 201 - 433 | 280.6 | 184 - 426 | 280.6 | 183 - 438 |  |
|  | Missing |  | 11 |  | 1 |  | 1 |  |  |
|  | coffee/infusions in 3rd trimester(g) |  | 200 | 99 - 286 | 200 | 46 - 279 | 200 | 29 - 286 |  |
|  | Missing |  | 3 |  | 0 |  | 1 |  | * |
|  | intake of calories in 3rd trimester(Kcal) |  | 1861 | 1579 - 2152 | 2092 | 1804 - 2460 | 2092 | 1818 - 2461 |  |
|  | Missing |  | 3 |  | 0 |  | 1 |  | ** |
|  | total fat in 3rd trimester(g) |  | 67.92 | 56 - 83 | 91.03 | 76 - 109 | 91.03 | 78 - 111 |  |
|  | Missing |  | 3 |  | 0 |  | 1 |  | ** |
|  | consumption of dairy  in 3rd trimester(g) |  | 509 | 327 - 650 | 525.3 | 323 - 647 | 525.3 | 311 - 647 |  |
|  | Missing |  | 3 |  | 0 |  | 1 |  |  |
|  | consumption of eggs  in 3rd trimester(g) |  | 21.62 | 22 - 22 | 21.62 | 22 - 22 | 21.62 | 22 - 22 |  |
|  | Missing |  | 3 |  | 0 |  | 1 |  | * |
|  | meat  in 3rd trimester(g) |  | 91.36 | 69 - 114 | 124.9 | 95 - 150 | 124.9 | 98 - 155 |  |
|  | Missing |  | 3 |  | 0 |  | 1 |  | ** |
|  | fish in 3rd trimester(g) |  | 69.33 | 53 - 89 | 63.37 | 44 - 91 | 63.37 | 43 - 91 |  |
|  | Missing |  | 3 |  | 0 |  | 1 |  | * |
|  | vegetables in 3rd trimester(g) |  | 196.9 | 150 - 262 | 212.8 | 147 - 280 | 212.8 | 147 - 276 |  |
|  | Missing |  | 3 |  | 0 |  | 1 |  |  |
|  | fruits in 3rd trimester(g) |  | 274.9 | 197 - 397 | 313.1 | 185 - 444 | 313.1 | 179 - 449 |  |
|  | Missing |  | 3 |  | 0 |  | 1 |  |  |
| **Clinical** | Cholesterol (mg/dl) |  | 190 | 171 - 213 | 187 | 170 - 207 | 187 | 169 - 208 |  |
|  | Missing |  | 3 |  | 13 |  | 11 |  |  |
|  | Triglycerides (mg/dl) |  | 90 | 68 - 115 | 100 | 77 - 129 | 100 | 77 - 130 |  |
|  | Missing |  | 3 |  | 13 |  | 11 |  | ** |
|  | Folic acid (µg/l) |  | 16.4 | 11-22 | 11.2 | 8.5 - 16 | 11.2 | 8.4 - 17 |  |
|  | Missing |  | 3 |  | 13 |  | 12 |  | ** |
|  | Vitamin B12 (pg/l) |  | 325 | 271 - 383 | 298 | 257 - 366 | 298 | 256 - 364 |  |
|  | Missing |  | 2 |  | 7 |  | 6 |  | ** |
|  | Vitamin D (ng/ml) |  | 27.9 | 22 - 37 | 72.6 | 51 - 91 | 72.6 | 53 - 93 |  |
|  | Missing |  | 3 |  | 7 |  | 9 |  | ** |
|  | Tsh mother (µU/ml) |  | 1.29 | 0.83 - 1.9 | 1.21 | 0.8 - 1.8 | 1.21 | 0.8 - 1.7 |  |
|  | Missing |  | 3 |  | 36 |  | 36 |  |  |
|  | T3 mother (nmol/L) |  | 2.4 | 2.2 - 2.6 | 2.385 | 2.2 - 2.6 | 2.385 | 2.2 - 2.7 |  |
|  | Missing |  | 3 |  | 36 |  | 37 |  |  |
|  | T4 mother (pmol/l) |  | 10.1 | 9.3 - 11 | 10.7 | 9.8 - 12 | 10.7 | 9.8 - 12 |  |
|  | Missing |  | 3 |  | 36 |  | 36 |  | ** |
|  | Protein C reactive (mg/dl) |  | 0.365 | 0.2 - 0.6 | 4 | 3 - 7.8 | 4 | 3 - 7.8 |  |
|  | Missing |  | 4 |  | 78 |  | 91 |  | ** |
|  | Iodine in 1st trimester (µg/l) |  | 176.5 | 112 - 288 | 101 | 62 - 160 | 101 | 55 - 154 |  |
|  | Missing |  | 0 |  | 231 |  | 261 |  | ** |
|  | Ferritin mother (µg/l) |  | 26.7 | 16 - 41 | 26.89 | 15 - 44 | 26.89 | 15 - 45 |  |
|  | Missing |  | 2 |  | 6 |  | 5 |  |  |
|  | Rate of weight gain during pregnancy | Recommended | 160 | 38.8 | 143 | 36.3 | 178 | 38.0 |  |
|  |  | Low | 90 | 21.8 | 82 | 20.8 | 92 | 19.6 |  |
|  |  | High | 131 | 31.8 | 160 | 40.6 | 187 | 39.9 |  |
|  | Missing |  | 31 | 7.5 | 9 | 2.3 | 12 | 2.6 |  |
|  | Gestational diabetes | None | 357 | 86.7 | 132 | 33.5 | 159 | 33.9 |  |
|  |  | Impaired glucose tolerance (IGT) | 15 | 3.6 | 62 | 15.7 | 79 | 16.8 | ** |
|  |  | Gestational diabetes mellitus (GDM) | 16 | 3.9 | 9 | 2.3 | 10 | 2.1 |  |
|  |  | Diabetes diagnosed prior to pregnancy (DM) | 2 | 0.5 | 0 | 0.0 | 0 | 0.0 |  |
|  |  | Gestational diabetes mellitus (GDM2) | 0 | 0.0 | 1 | 0.3 | 2 | 0.4 |  |
|  | Missing |  | 22 | 5.3 | 190 | 48.2 | 219 | 46.7 |  |
| **Other** | Season of birth | Spring | 119 | 28.9 | 113 | 28.7 | 129 | 27.5 |  |
|  |  | Summer | 139 | 33.7 | 98 | 24.9 | 152 | 32.4 |  |
|  |  | Fall | 90 | 21.8 | 101 | 25.6 | 117 | 24.9 |  |
|  |  | Winter | 64 | 15.5 | 82 | 20.8 | 71 | 15.1 |  |
|  | Missing |  | 0 |  | 0 |  | 0 |  |  |
|  |  |  |  |  |  |  |  |  |  |
