# Supplementary material for: Maternal urinary metabolic signatures of fetal growth and associated clinical and environmental factors in the INMA study
Source: BMC Med. 2016 Nov 4;14:177. doi: 10.1186/s12916-016-0706-3 (PMC5097405; doi:10.1186/s12916-016-0706-3)
Supplement: Additional file 8: Table S3. — Decomposition of variance in Birthweight. (DOCX 41 kb) [file 12916_2016_706_MOESM8_ESM.docx]

## Additional file 8: Table S3: Decomposition of variance in Birthweight

Table 3-1 Multiple regression analysis of factors affecting birth WEIGHT in two cohorts, Gipuzkoa and Sabadell (variance decomposition)

|  | Gipuzkoa (n=298) | Sabadell (n=207) |
| --- | --- | --- |
| Panel metabolites at 34 wk (n=10) | 12% | 12% |
| Constitutional birth weight determinants (n=7) | 64% | 55% |
| Clinical biochemistry (n=11) | 10% | 13% |
| Diet at 34 wk (n=9) | 4% | 3% |
| Lifestyle/Exposure (n=11) | 5% | 13% |
| Socio-demographic (n=7) | 3% | 4% |
| **Total variance explained (Adjusted R^2^)** | **56%** | **47%** |

TABLE 3-2 Detailed table with each individual parameter imputed in the model to explain birth weight:

|  | Sabadell | | Gipuzkoa | |
| --- | --- | --- | --- | --- |
|  | % Adjusted R^2^ | p-values | % Adjusted R^2^ | p-values |
| Progesterone metabolite | 0.4% | 0.512 | 1.4% | 0.180 |
| Pregnanediol-3-G | 0.2% | 0.620 | 0.0% | 0.806 |
| Oestrogen metabolite | **6.3%** | **0.008** | 0.3% | 0.547 |
| Leucine | 0.5% | 0.467 | 0.1% | 0.728 |
| Isoleucine | 2.3% | 0.110 | 2.3% | 0.081 |
| Valine | 0.2% | 0.621 | 2.3% | 0.079 |
| 3-hydroxyisobutyrate | 0.6% | 0.418 | 0.0% | 0.988 |
| 3-hydroxybutyrate/3-aminoisobutyrate | 0.0% | 0.920 | 0.4% | 0.483 |
| Alanine | 0.7% | 0.389 | **5.1%** | **0.009** |
| Choline | 0.4% | 0.517 | 0.0% | 0.852 |
| Mother weight pre-pregnancy | **5.1%** | **0.018** | **8.0%** | **0.001** |
| Mother height | 3.4% | 0.054 | **11.5%** | **7.86E-05** |
| Father height | 3.3% | 0.057 | 1.9% | 0.113 |
| Parity | 1.7% | 0.175 | 2.0% | 0.100 |
| gestational age 34 | 0.7% | 0.392 | 2.4% | 0.077 |
| gestational age birth | **37.6%** | **2.18E-11** | **35.6%** | **4.94E-13** |
| Newborn gender | 2.9% | 0.077 | 2.6% | 0.064 |
| Cholesterol (mg/dl) | 0.1% | 0.699 | 0.9% | 0.276 |
| Triglycerides (mg/dl) | 0.4% | 0.536 | 1.4% | 0.175 |
| Folic acid (µg/l) | 0.2% | 0.680 | 0.1% | 0.785 |
| Vitamin B12 (pg/l) | 0.6% | 0.415 | 1.4% | 0.180 |
| Vitamin D (nanograms/ml) | 0.2% | 0.652 | 0.3% | 0.518 |
| Tsh mother (microU/ml) | 3.1% | 0.067 | 0.1% | 0.700 |
| T3 mother (nmol/L) | 0.1% | 0.770 | 0.1% | 0.698 |
| T4 mother (pmol/l) | 0.7% | 0.397 | 0.2% | 0.572 |
| Protein C reactive (mg/dl) | 0.0% | 0.974 | 0.2% | 0.598 |
| Ferritine mother (µg/l) | 0.3% | 0.577 | 0.0% | 0.979 |
| Rate of weight gain during pregnancy (IOM classification) | **7.8%** | **0.003** | **5.0%** | **0.010** |
| coffee/infusions at week 32(g) | 0.0% | 0.827 | 2.1% | 0.094 |
| intake of calories at week 32(Kcal) | 0.7% | 0.396 | 0.0% | 0.904 |
| total fat at week 32(g) | 1.1% | 0.266 | 0.8% | 0.321 |
| consumption of dairy  at week 32(g) | 0.0% | 0.875 | 0.3% | 0.526 |
| consumption of eggs  at week 32(g) | 0.3% | 0.541 | 0.6% | 0.384 |
| meat  at week 32(g) | 0.4% | 0.537 | 0.0% | 0.873 |
| fish at week 32(g) | 0.0% | 0.867 | 0.1% | 0.745 |
| vegetables at week 32(g) | 0.2% | 0.660 | 2.1% | 0.094 |
| fruits at week 32(g) | 0.2% | 0.628 | 0.2% | 0.644 |
| Hours of sleep at week 32 | 0.0% | 0.945 | 0.5% | 0.438 |
| Overall physical activity at week32 | 0.0% | 0.831 | 0.6% | 0.370 |
| Night work | 0.0% | 0.864 | 1.0% | 0.250 |
| Main source of drinking water at home | **5.6%** | **0.013** | 0.2% | 0.605 |
| Main source of drinking water outside home | 0.4% | 0.499 | 0.5% | 0.428 |
| Passive smoking | 1.1% | 0.275 | 1.1% | 0.223 |
| Average daily intake of alcohol (g) at week 32 | 0.1% | 0.762 | 0.2% | 0.598 |
| Smoking before pregnancy (cigarettes/day) | 3.2% | 0.060 | 0.3% | 0.564 |
| Smoking at the beginning of pregnancy (cigarettes/day) | 1.8% | 0.162 | 0.3% | 0.553 |
| Smoking at week 32 of pregnancy (cigarettes/day) | 0.2% | 0.648 | 0.2% | 0.571 |
| Season at birth | 0.4% | 0.510 | 0.0% | 0.872 |
| Mother age at last menstrual period adjusted | 0.3% | 0.579 | 0.1% | 0.792 |
| Mother ethnicity | 0.3% | 0.543 | 0.1% | 0.691 |
| Country of birth: mother | 1.4% | 0.222 | 0.0% | 0.804 |
| Maternal social class (3categories),based on CNO-94 | 0.1% | 0.743 | 0.3% | 0.536 |
| Paternal social class(3categories),based on CNO-94 | 1.7% | 0.176 | 1.6% | 0.144 |
| Maternal education | 0.4% | 0.487 | 0.6% | 0.372 |
| Paternal education | 0.2% | 0.611 | 0.6% | 0.364 |
| Total | 100% |  | 100% |  |

{Bibliography}

1. Iñiguez C, Ballester F, Costa O, Murcia M, Souto A, Santa-Marina L, et al. Maternal smoking during pregnancy and fetal biometry: the INMA Mother and Child Cohort Study. Am J Epidemiol. 2013 Oct 1;178(7):1067–75.

2. Stanley EG, Bailey NJC, Bollard ME, Haselden JN, Waterfield CJ, Holmes E, et al. Sexual dimorphism in urinary metabolite profiles of Han Wistar rats revealed by nuclear-magnetic-resonance-based metabonomics. Anal Biochem. 2005 Aug 15;343(2):195–202.

3. Epskamp S, Cramer AOJ, Waldorp LJ, Schmittmann VD, Borsboom D. qgraph : Network Visualizations of Relationships in Psychometric Data. J Stat Softw. 2012;48:1–18.
