# Supplementary material for: Maternal urinary metabolic signatures of fetal growth and associated clinical and environmental factors in the INMA study
Source: BMC Med. 2016 Nov 4;14:177. doi: 10.1186/s12916-016-0706-3 (PMC5097405; doi:10.1186/s12916-016-0706-3)
Supplement: Additional file 9: Figure S5. — Potential sources of variation in metabolite signatures of fetal growth in 1st trimester from epidemiological data such as lifestyle and clinical parameters in Sabadell. (PDF 93 kb) [file 12916_2016_706_MOESM9_ESM.pdf]

SUPPORTING INFORMATION FIGURE 5: DECOMPOSITION OF VARIANCE EXPLAINED IN FIRST TRIMESTER METABOLIC PHENOTYPES IN SABADELL BASED ON CLINICAL AND LIFESTYLE FACTORS

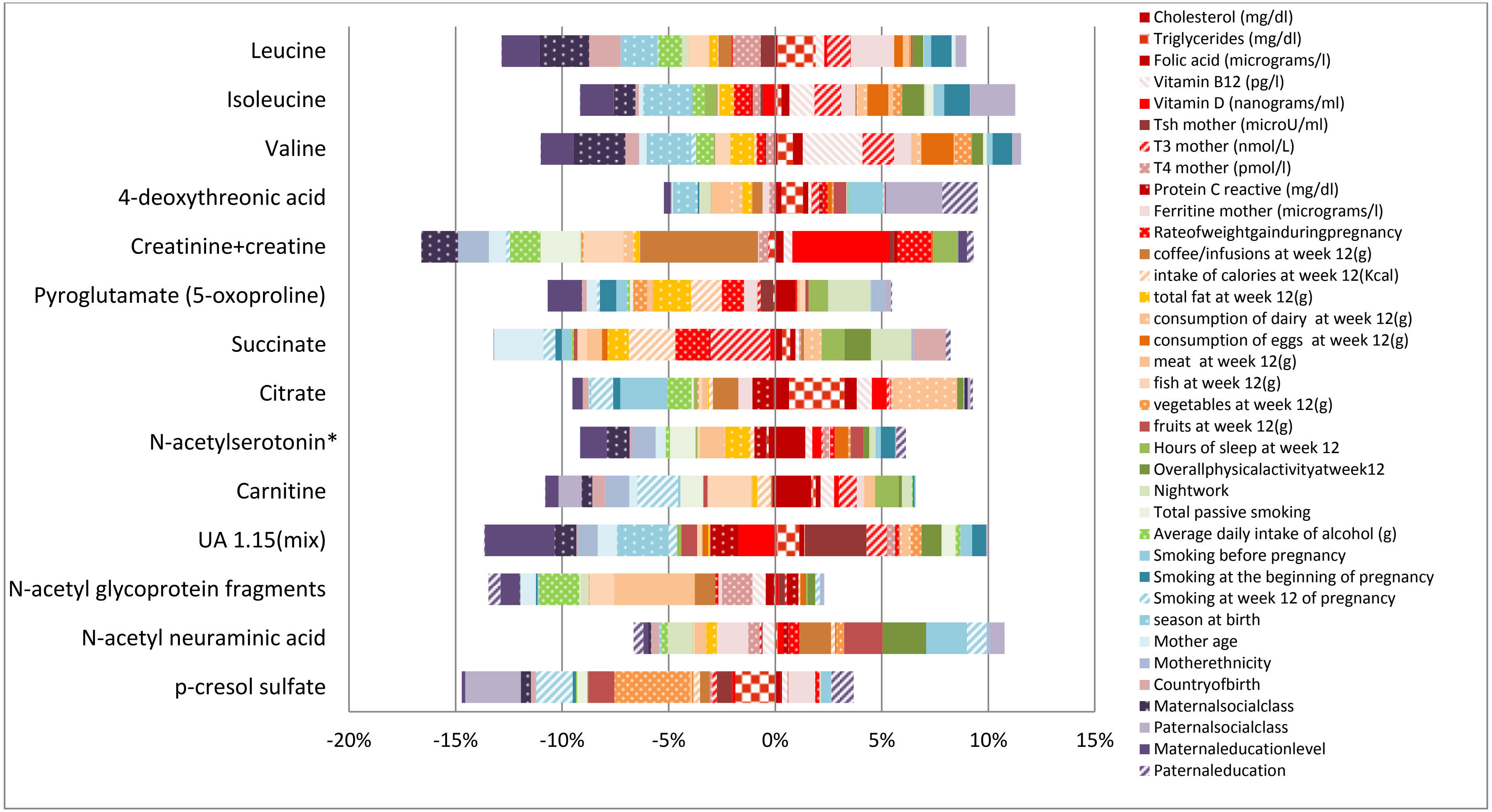

*tentative assignment
